# Activation of GPER1 in macrophages ameliorates UUO-induced renal fibrosis

**DOI:** 10.1038/s41419-023-06338-2

**Published:** 2023-12-12

**Authors:** Lin Xie, Ye Cheng, Wen Du, Lili Fu, Zhaonan Wei, Yuting Guan, Yi Wang, Changlin Mei, Chuanming Hao, Min Chen, Xiangchen Gu

**Affiliations:** 1 Department of Nephrology, Yueyang Hospital of Integrated Traditional Chinese and Western Medicine, Shanghai University of Traditional Chinese Medicine, Shanghai, 200437 China; 2 Department of Nephrology, Ruijin Hospital, Shanghai Jiaotong University School of Medicine, Shanghai, 200025 China; 3 Department of Nephrology, Huashan Hospital, Fudan University, Shanghai, 200040 China; 4 Department of Nephrology, Changzheng Hospital, Naval Medical University, Shanghai, 200001 China; 5 Shanghai Frontiers Science Center of Genome Editing and Cell Therapy, Shanghai Key Laboratory of Regulatory Biology, Institute of Biomedical Sciences and School of Life Sciences, East China Normal University, Shanghai, 200241 China; 6 Department of Medicine, Shanghai Hospital of Civil Aviation Administration of China, Shanghai, 201201 China

**Keywords:** Translational research, End-stage renal disease

## Abstract

Numerous studies have proven the critical role of macrophages in the renal fibrosis process. Notably, G Protein-coupled Estrogen Receptor 1 (GPER1), a novel estrogen receptor, has been shown to play a ubiquitous role in regulating macrophage activities and proinflammatory pathways. However, the precise role of GPER1 in macrophage-mediated renal fibrosis is unknown. In this study, we aimed to investigate the function of macrophage GPER1 in the UUO-induced renal fibrosis model. Compared to vehicle-treated ovariectomized (OVX) female and male unilateral ureteral obstruction (UUO) models, we observed that G-1 (GPER1 agonist)-treated OVX female and male UUO mice had fewer renal fibrotic lesions and less M1 and M2 macrophage infiltration in the kidney tissues. Conversely, *Gper1* deletion in male UUO mice accelerated renal fibrosis and increased inflammation. In vitro studies also revealed that GPER1 activation reduced M0 macrophage polarization towards M1 or M2 phenotypes. The RNA-sequencing analysis and immunoblotting indicated that GPER1 activation was primarily involved in downregulating immune pathways activation and inactivating MAPK pathways. Tubular epithelial cells co-cultured with G-1-pretreated M1 macrophages exhibited fewer injuries and immune activation. In addition, fibroblasts co-cultured with G-1-pretreated M2 macrophages showed downregulated extracellular matrix expression. Overall, this is the first study to demonstrate the effect of GPER1 on macrophage-mediated renal fibrosis via inhibition of M1 and M2 macrophage activation. These findings indicate that GPER1 may be a promising therapeutic target for treating renal fibrosis.

## Introduction

Chronic kidney disease (CKD) is a worldwide health concern affecting ~10% of the adult population [1]. Renal fibrosis is regarded as the final common pathway of CKD, ultimately leading to end-stage renal disease. Unfortunately, current treatments for renal fibrosis prevention are inefficient; hence, effective therapies are urgently needed.

Estradiol has been shown to protect against renal injuries [2–4]. However, the side effects of estradiol limit its applicability in clinical practice, and the associated mechanism is not fully understood either. It is known that the classical estrogen receptors, ERα and ERβ, do not account for all protective effects of estrogen [5]. This resulted in the discovery of a novel estrogen receptor, GPER1, which is expressed in multiple organs and is involved in various biological processes. Recent studies have demonstrated that GPER1 is crucial in the immune response and inflammation, indicating its potential as a therapeutic target [6]. Our previous study also showed that antagonizing GPER1 exacerbated renal fibrosis in the UUO model, but the mechanism remains unknown [7].

Macrophages have attracted great attention in the progression of kidney injury, inflammation, and renal fibrosis [8], whereas activation of GPER1 exerts anti-inflammatory effects via actions on macrophages. Numerous studies have demonstrated that GPER1 mediates the anti-inflammatory effect of estrogen in the monocyte/macrophage population via several mechanisms [9]. In particular, G-1 (GPER1 agonist) has been shown to decrease TLR4 expression in mouse macrophage cell lines and peritoneal macrophages [9]. Moreover, treating mouse macrophage cell lines with G-1 reduces the inflammatory response to LPS [10]. These findings suggest that GPER1 activation may protect against renal fibrosis by inhibiting inflammation in macrophages. Wei et al. [11] observed that *Gper1* deletion mice increased hepatocarcinogenesis in the HCC model. In vitro study revealed that IL-6 was downregulated in a GPER1-dependent manner in the BMDMs isolated from wild-type and mutant mice [11]. These findings confirmed the role of GPER1 in modulating the inflammatory response and implied that GPER1 might play a similar role in the renal fibrosis process.

In this study, we sought to investigate the role of GPER1 in macrophages and its potential effects on renal fibrosis progression and development.

## Materials and methods

### Animal experiments

All animal experiments were reviewed and authorized by the Institutional Animal Care and Use Committee of the Shanghai University of Traditional Chinese Medicine. This was done following the institutional guidelines before the experiment. The *Gper1* mutant mice were generated by BRL Company Shanghai, China, in accordance with institutional protocols by co-injecting Cas9 mRNA (100 ng/L; Thermo Fisher) and sgRNA (50 ng/L). Using the Guide-it TM sgRNA In Vitro Transcription Kit from Takara, two sgRNAs were produced. The genotyping primers and sgRNA sequences are shown in Supplementary Table 1. After breeding these *Gper1* knockout mice for more than three generations, they were verified by genotyping toe DNA using PCR. To avoid confounding effects of age and strain background, littermate controls were used for all phenotypic analyses of genetically modified mouse lines. C57BL/6 wild-type mice were obtained from JSJ Co., Ltd (Shanghai, China). Three types of operations, including Ovariectomy (OVX), mini-pump implantation, and unilateral ureteral obstruction (UUO) were performed on female mice. Two types of operations(mini-pump implantation [12], UUO) were performed on male mice. For each surgery, the mice were anesthetized with 2% pentobarbital (40 mg/kg) and placed on a heating pad. Also, 40 ml/kg 0.9% saline was subcutaneously injected during the surgery. For pain control, mice were given buprenorphine subcutaneously after surgery. Ovariectomy (OVX) [13] was performed on 8-week-old wild-type female mice seven days before mini-pump implantation. Dorsolateral incisions were made, and ovaries were removed by pulling out. On the other hand, mice in the sham group received the same surgery without the removal of ovaries. The G-1 (Cayman Chemical, US) was solubilized in DMSO before use. Minipumps (Alzet,1007D, Durect, CA, USA) infused with G-1 were subcutaneously implanted into mice at the rate of 200 ug/ kg/d. The vehicle group received minipumps infused with DMSO without G-1. UUO surgeries were performed on male and female mice after three weeks of mini-pump implantation. As previously described [14], the left kidney was externalized after a brief incision through the left flank muscle. The left ureter was then wrapped in a surgical knot with a 4-0 silk suture near the base of the left kidney. The right kidney was exposed without wrapping the surgical knots on the right ureter. Seven days after the operation, mice were operated on to remove the contralateral and obstructed kidneys. For RNA and protein extraction, paraffin embedding, or flow cytometry, kidney slices were collected.

### Cell culture

#### Cell lines and culture conditions

The L929 cells were cultured in high-glucose DMEM with 10% FBS. The supernatant containing macrophage colony-stimulating factor secreted by the L929 cells was collected and filtered using a 0.22 μm filter.

### Primary tubular epithelial cell culture

As previously described [15], kidneys were first digested with 2 mg/ml type I collagenase (Thermo Fisher Scientific). Then the cell suspensions were filtered through 100 μm, 70 μm, and 40 μm cell strainers, followed by culturing the tubular epithelial cells (TECs) pellet in RPMI 1640 medium with 10% FBS containing 50 ng/ml EGF (Gibco).

### Bone marrow-derived macrophage isolation and culture

BMDMs were isolated from wild-type and *Gper1*^*−/−*^ mice to perform the cellular experiment. Bone marrow was flushed from the femur and tibia, then seeded at a concentration of 1 × 10^6^/ml and cultured in the media containing RPMI 1640 (Lonza), 20% L929 cell culture supernatant, 15% FBS and 1% Penicillin & Streptomycin. Media were refreshed on days one and four. Differentiated BMDMs on day 7 were treated with LPS (100 ng/ml, Sigma) and 20 ng/ml INF-γ (Peprotech) or 20 ng/ml IL-4 (Peprotech) for M1 or M2 macrophage phenotype stimulation, respectively. G-1 at a concentration of 10 μmol/L dissolved in DMSO was also used for GPER1 activation.

### Primary mouse fibroblast culture

Primary mouse fibroblasts were isolated from the kidneys of wild-type mice, as previously described [14]. Wild-type 6–8-week-old mice were anesthetized and perfused with PBS. The kidneys were then decapsulated and cut into 1–3 mm^3^ pieces, seeded in a 10 cm plate, and grown in high-glucose DMEM with 20% FBS. The remaining tissues were discarded after the cells outgrew the explant. After 1–2 passages, the cells were co-cultured with macrophages in the trans-well system.

### ELISA

The BMDMs were stimulated with G-1 (10 μmol/L) or DMSO for 48 h with LPS/INF-γ. The levels of IL-6, G-CSF, and TNF-α in the supernatant of M1 macrophages were quantified using the Enzyme-linked immunosorbent assay kits following the instructions(Raybiotech). The concentration of each cytokine was defined by the absorbance measured with a microplate reader at 450 nm.

### Real-time PCR

The RNA was extracted from kidney tissue or cultured cells using RNA extraction kits (Vazyme). Reverse transcription kits (Takara) were used to obtain cDNA. Amplified cDNA was used as a template for quantitative PCR. The mRNA expression was measured by quantitative RT-PCR using ABI 7500 with SYBR green master mix (Vazyme), and the specific primers are presented in Supplementary Table 2. The relative expressions of gene transcript were calculated as 2^−Δ target gene CT value -housekeeping gene CT value^.

### Histology and immunohistochemical staining

Patients’ informed consent was obtained from each participant for kidney sample staining, and the study was approved by the Human Research Ethics Committee of Yueyang Hospital. After fixation with 4% paraformaldehyde and embedding in paraffin, the kidney samples were sliced into 4 μm-thick serial sections. After deparaffinization, the sections were stained with hematoxylin/eosin, periodic acid-Schiff, and Masson’s Trichrome. In addition, the deparaffinized sections were incubated overnight at 4 °C with primary antibodies listed in Supplementary Table 3, followed by incubation with secondary antibodies combined with streptavidin-HRP using the ABC method. Slides were observed, and images were taken using an Olympus microscope (BX53). The staining intensity was quantified using ImageJ software in ten randomly chosen nonoverlapping fields (200 magnification) [16].

### Western blot

Proteins were extracted from the kidneys and cells using RIPA lysis buffer containing the phosphatase inhibitor and proteinase inhibitor cocktail (Roche). The protein concentrations were measured and balanced using the Pierce™ BCA Protein Assay Kit. Equal amounts of protein lysates were separated on 7.5% or 10% SDS-PAGE gel, respectively, and then electro-transferred to PVDF membranes. The membranes were incubated overnight with primary antibodies shown in Supplementary Table 3 at 4 °C overnight and subsequently with the secondary antibodies at room temperature for 1 h. Images of bands were captured using a chemiluminescence imaging system, and the densities of the bands were analyzed by ImageJ software.

### Immunofluorescence staining

The sections were deparaffinized, and endogenous peroxidase was then inactivated. After blocking, the slides were immune-stained with the primary antibodies (Supplementary Table 4) at 4 °C overnight. This was followed by washing and incubation with a fluorophore-linked secondary antibody. Slides were viewed with an Olympus Epi-fluorescence microscope equipped with a digital camera (BX53).

### Flow cytometry

Macrophage populations were identified using multiparametric flow cytometry. To isolate the cells, sliced kidneys were incubated in serum-free DMEM containing 0.5 mg/mL of Roche Basel’s Liberase DL and 100 U/mL of Roche Basel’s DNase for 30 min, followed by filtration through a 40 μm filter. The cells were incubated with the specific fluorochrome-conjugated antibodies shown in Supplementary Table 4. In addition, flow minus one control for each fluorophore was performed to establish gates. The FlowJo software was used to analyze data. At least 20,000 singlets from each kidney sample were examined in triplicate. The forward versus side scatter (FSC vs. SSC) plot was initially used as a basis for cell gating. The presence of CD45 and CD11b, which are expressed in inflammatory and hematopoietic cells, in addition to F4/80, a distinctive macrophage surface marker, was used to identify the macrophage cell population. The M1 and M2 macrophage subpopulations have been determined using CD86 and CD206, respectively.

### Cell viability

Primary BMDMs were seeded evenly into a 96-well plate. Each well was treated with DMSO, or G-1 (10 μmol/L, Cayman), or LPS (100 ng/ml, Sigma) + INFγ (20 ng/ml, Peprotech) or LPS + INFγ + G-1 for 24 h in the incubator. Then 10 μl of CCK8 solution was added to each plate well and incubated for 4 h. The absorbance rate at 450 nm was measured and calculated by the microplate reader.

### Co-culture trans-well assay

After stimulating with LPS (100 ng/ml) and 20 ng/ml Interferon-γ (INFγ) or 20 ng/ml IL-4 with or without 10 μmol/L G-1 for 48 h, BMDMs seeded in the lower chamber were vigorously rinsed with PBS thrice to avoid the transfer of the medium with LPS, INFγ, IL-4, or G-1 to the upper chambers. Using 0.4 μm pore-sized trans-wells, the BMDMs in the lower chambers were co-cultured with PTECs or primary fibroblasts from the different upper chambers for another 24 h. The PTECs or primary fibroblasts from the upper chambers were harvested for RNA and protein extraction.

### RNA-sequencing (RNA-seq) and bioinformatics analysis

Total cellular RNA was extracted from the cells (Seq1: BMDMs, BMDMs after G-1 for 48 h treatment; Seq2: BMDMs after 48 h of LPS and INFγ treatment, BMDMs after 48 h of LPS and INFγ and G-1 treatment; Seq3: BMDMs after 48 h of IL-4 treatment, BMDMs after 48 h of IL-4 and G-1 stimulation; Seq4: TECs after 24 h co-cultured with M1 BMDMs, TECs after 24 h of co-culture with G-1 pretreated M1 BMDMs were extracted using TRIzol reagent (15596018, Invitrogen) (*n* = 3). Three samples in each group were pooled to yield one sample for RNA sequencing. The purity and quantity of each mRNA sample were examined using the Nanodrop 2000 spectrophotometer (Thermo Scientific, USA). RNA libraries were constructed using the VAHTS® Universal V6 RNA-seq Library Prep Kit. Transcriptome sequencing and analysis were conducted by OE Biotech Co., Ltd (Shanghai, China), and paired-end reads were obtained on the Novaseq 6000 platform. The quality of RNA-seq data was estimated using RSeQC (version 2.6.4). Differentially expressed genes (DEGs) were identified using the DESeq2. *P* value < 0.05 and foldchange >2 or foldchange <0.5 was set as the threshold for selecting significant DEGs. Based on the hypergeometric distribution, gene ontology (GO) enrichment analysis of DEGs was performed to select the significantly enriched genes using David bioinformatics. RNA sequencing raw data and processed data were submitted to NCBI GEO, and the GEO accession number is GSE235718.

### Statistical analyses

Data are presented as Mean ± SEM. The data were assessed for Gaussian distribution and homogeneity of variances before further analysis. Log transformation was utilized when the variances were not sufficiently homogeneous. Statistical significance was determined using an unpaired *t* test and two-way ANOVA with Bonferroni correction for multiple comparisons (GraphPad Software). Statistical significance was presented by asterisks (*P < 0.05, **P < 0.01, ***P < 0.001, ****P < 0.0001).

## Results

### GPER1 is expressed in macrophages of renal tissues in CKD patients and UUO model mice

**Fig. 1:**
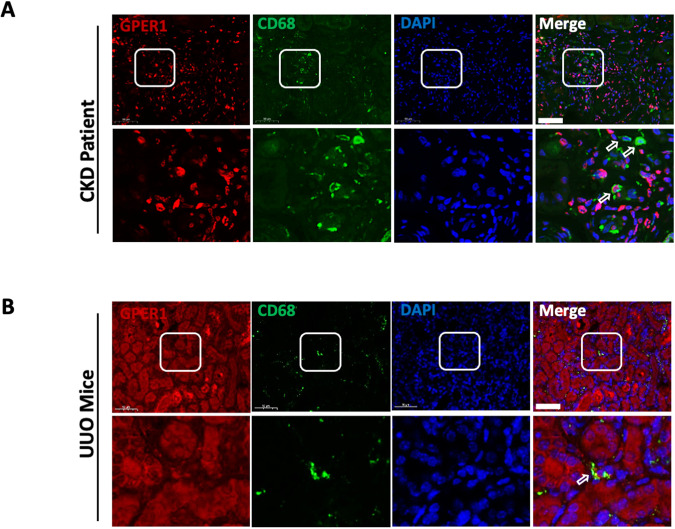
GPER1 is colocalized with CD68-positive cells in CKD patients and UUO model mice. **A** Representative images of GPER1 and CD68 immunostaining from kidney tissues of CKD patients. [GPER1 (red), CD68 (green) and DAPI(blue)]. **B** Representative images of GPER1 (red) and CD68 (green) immunostaining from obstructed kidney tissues of OVX mice. CD68 is the marker of macrophages. Arrows mark the colocalization of GPER1 and CD68. Scale bars: 50 μm.

To identify whether GPER1 is expressed in the macrophages of the kidney, we first co-stained GPER1 together with macrophage marker CD68 in the renal tissues of CKD patients. The GPER1 was found to be colocalized with CD68-positive cells (Fig. 1A). Similarly, CD68-positive cells co-stained with GPER1 were observed in the kidneys of UUO model mice (Fig. 1B). These data indicate that GPER1 expressed in macrophages may involve in renal fibrosis development in CKD patients and mice.

### Activation of GPER1 in OVX female mice attenuates UUO-induced renal fibrosis

**Fig. 2:**
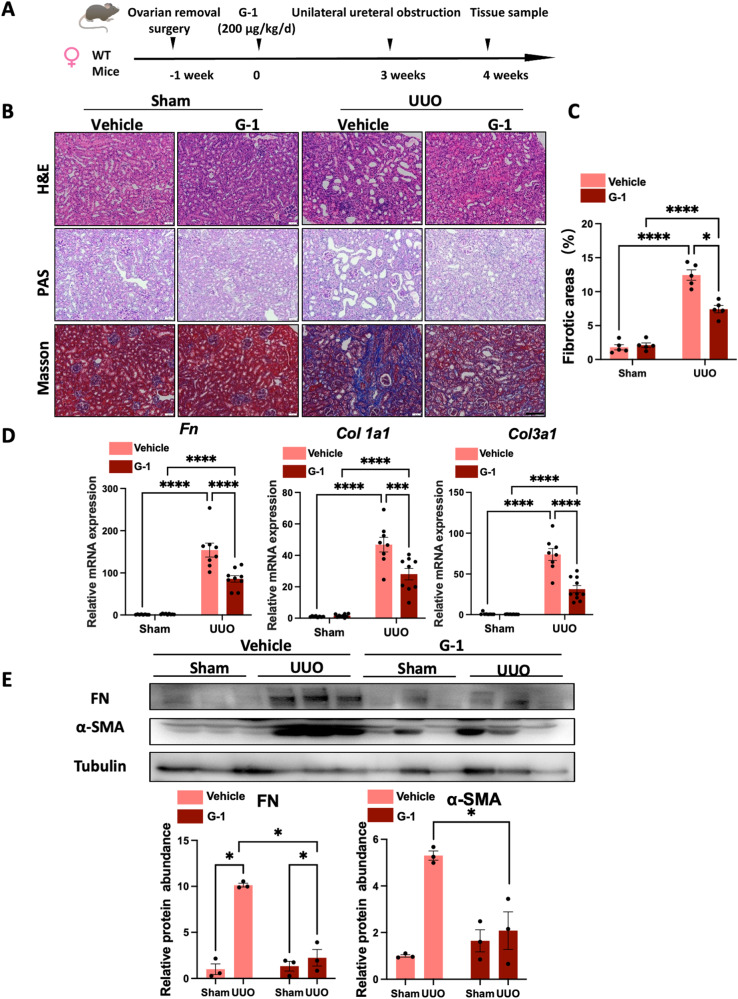
GPER1 activation attenuates UUO-induced renal fibrosis in OVX female mice. **A** Schematic diagram shows the experimental design. **B** Representative images of kidney sections of H&E, Masson’s trichrome, and PAS staining from OVX female mice subjected to UUO with or without G-1 treatment for 7 days. Scale bars: 100 μm. **C** Quantification of Masson’s trichrome staining (*n* = 5 per group). **D** Relative mRNA expression for *Fn, Col1a1, Col3a1* in sham and obstructed kidneys from Vehicle or G-1 treated OVX female mice (*n* = 8 in the Vehicle group, *n* = 9 in the G-1 treated group). **E** Representative immunoblots of Fibronectin and α-SMA in sham and obstructed kidney tissues of OVX mice treated with or without G-1 (*n* = 3). Densitometry analysis was performed to quantify protein expression. Data are shown as means ± SEM. Statistical analysis by two-way ANOVA with Tukey’s test. *P < 0.05,**P < 0.01, ***P < 0.001, ****P < 0.0001.

As previously described [11], GPER1 is a de novo estrogen receptor like the estrogen receptors α and β. Many studies have shown estrogen binding to GPER1 and activating rapid non-genomic signaling [11]. To rule out the possibility of estrogen affecting the GPER1, we performed ovariectomy on the 8-week-old female mice. Subsequently, OVX mice were randomly divided into two groups treated with GPER1 agonist G-1 or vehicle for an additional 4 weeks. Then, UUO surgery was performed on mice after three weeks of G-1 treatment (Fig. 2A).

To evaluate the extent of kidney injury, kidney sections were stained with PAS and H&E staining (Fig. 2B). As expected, at seven days of UUO, the vehicle-treated OVX UUO model exhibited severe tubule-interstitial injuries, including the loss of brush border, tubular dilation, and immune cell infiltrations. These renal lesions were significantly reduced in OVX UUO mice treated with G-1 (Fig. 2B). Quantification of Masson’s trichrome staining indicated that, compared to vehicle-treated OVX UUO mice, G-1-treated OVX UUO mice displayed less extracellular matrix deposition in the kidney tissues (Fig. 2B, C). These findings were further supported by gene expression analysis, which showed the significant upregulation of *Col1a1*, *Col3a1*, and *Fn* in the vehicle-treated mice. In contrast, the administration of G-1 significantly suppressed *Col1a1*, *Col3a1*, and *Fn* mRNA expression (Fig. 2D). In addition, these results were also confirmed on protein levels by immunoblots of Fibronectin and α-SMA (Fig. 2E). Altogether, these data revealed that activation of GPER1 significantly attenuated renal fibrosis in the OVX UUO model.

### Activation of GPER1 downregulates M1 and M2 macrophage infiltrations in UUO murine OVX model

**Fig. 3:**
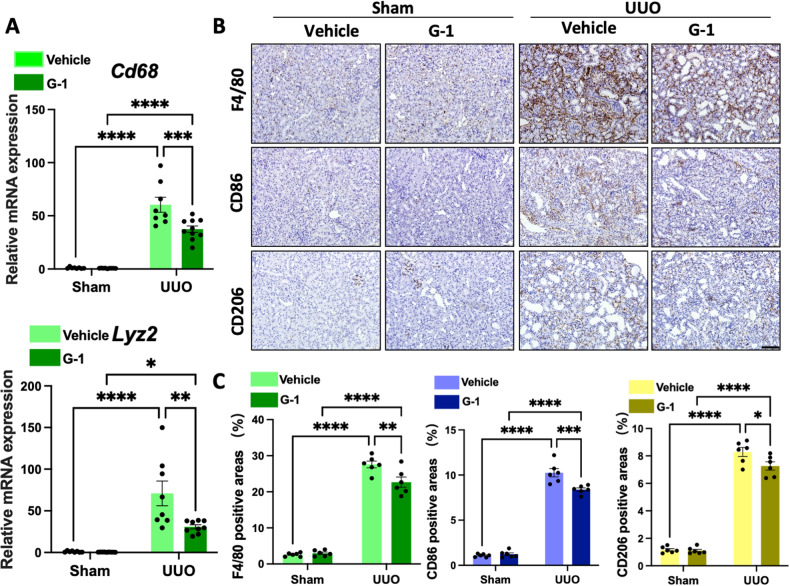
GPER1 inhibits M1 and M2 macrophage infiltrations in the kidney of the UUO model. **A** Quantitative RT-PCR of mRNA for macrophage *Cd68* and *Lyz2* in sham and obstructed kidneys from OVX female mice treated with or without G-1 (*n* = 8 in the Vehicle-treated group, *n* = 9 in the G-1-treated group). **B** Representative images of macrophage (F4/80), M1 macrophage (CD86), and M2 macrophage (CD206) immunohistochemistry-stained kidney samples of female OVX mice treated with or without G-1 treatment. Scale bars: 100 μm. **C** Quantification of F4/80, CD86, and CD206 immunohistochemistry staining (*n* = 6 in each group). Data are shown as means ± SEM. Two-way ANOVA with Tukey’s test. *P < 0.05,**P < 0.01, ***P < 0.001, ****P < 0.0001.

To further investigate whether GPER1 activation could affect macrophage infiltrations in the kidneys of the UUO model, we compared *Cd68* and *Lyz2* (the gene that encodes F4/80) transcript levels between the two groups. The result indicated that *Cd68* and *Lyz2* mRNA were significantly downregulated in the G-1-treated OVX UUO mice compared to the vehicle-treated OVX UUO mice (Fig. 3A). This data revealed that G-1 treatment reduced macrophage accumulation in the injured renal tissues. As quantified, F4/80 staining confirmed a significant reduction in macrophage infiltration in the kidneys of OVX UUO mice with G-1 administration versus the vehicle-treated OVX UUO mice (Fig. 3B, C).

Subsequently, we examined the two classical subsets of macrophages in the renal tissues of the UUO model with or without G-1 treatment. Activated macrophages are divided into two classical categories, M1 and M2 macrophages. The M1 macrophages are essential in promoting immune responses, while the M2 macrophage promotes fibrosis by secreting profibrotic growth factors [17, 18]. The M1 macrophage polarization is characterized by the upregulation of cell surface activation markers and molecules, like CD86 and M1 macrophage-associated proinflammatory cytokines, including tumor necrosis factor-α(*Tnf-a)*, Interleukin-1β (*Il-1b)*, Interleukin-6 (*Il-6)*, and nitric oxide synthase 2 (*Nos2*) [19]. Arginase1 (*Arg-1*), *Cd206(Mrc1)*, and Interleukin 10(*Il-10*) [18] are markers of M2 macrophage polarization.

**Fig. 4:**
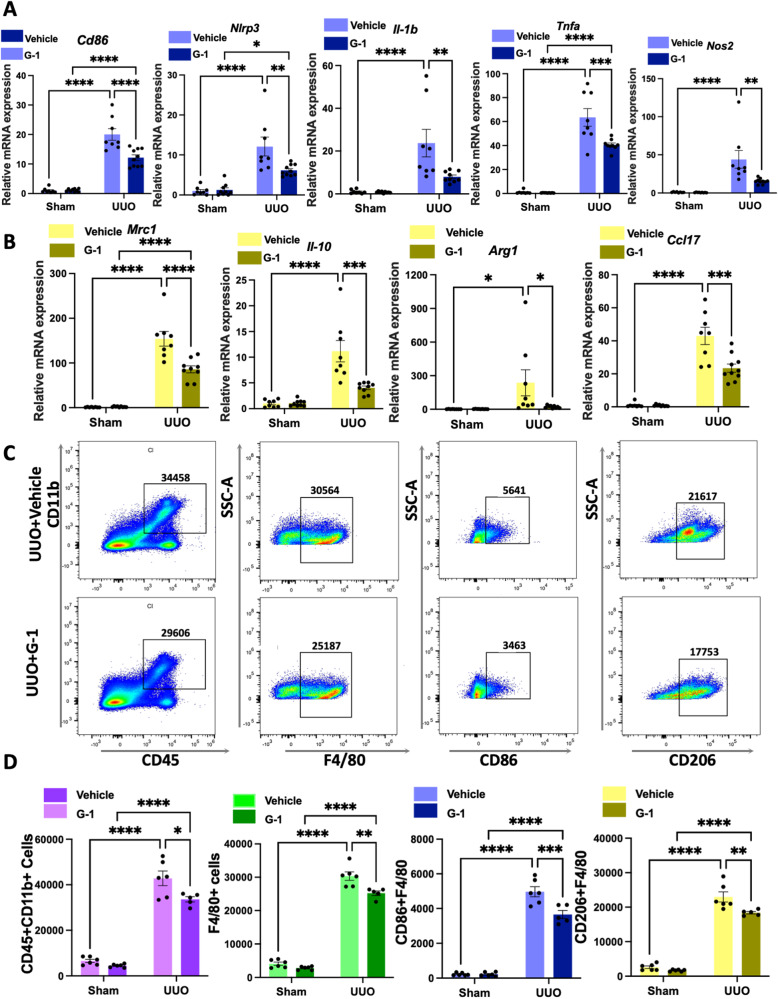
GPER1 attenuates M1 and M2 macrophage infiltration in the kidneys of OVX mice. **A** Relative mRNA expression of M1 macrophage-associated proinflammatory genes *Cd86, Nlrp3*, *Il-1b*, *Tnf-a*, and *Nos2* of the sham or obstructed kidney samples of OVX mice treated with or without G-1 (*n* = 8 in the Vehicle-treated group, *n* = 9 in the G-1-treated group). **B** Relative mRNA expression of M2 macrophage-associated genes *Mrc1*, *Il-10*, *Arg-1*, and *Ccl17* of the sham or obstructed kidney samples of mice treated with or without G-1 (*n* = 8 in the Vehicle-treated group, *n* = 9 in the G-1-treated group). **C** Representative flow cytometry dot plots of the expression of macrophages in kidneys from UUO OVX female mice with or without G-1 treatment. **D** Representative flow cytometric data and analysis of the counts of myeloid cells (CD45 + CD11b+), total macrophages (F4/80+), M1 macrophages (F4/80+, CD86+), M2 macrophages (F4/80+, CD206+) (*n* = 6 in the Vehicle-treated group, *n* = 5 in the G-1-treated group). Data are shown as means ± SEM. Two-way ANOVA with Tukey’s test. *P < 0.05,**P < 0.01, ***P < 0.001, ****P < 0.0001.

First, using qPCR, we analyzed proinflammatory gene expression and found a significant decrease in *Cd86*, *Nlrp3*, *Il-1β*, *Tnf-a*, and *Nos2* expression in the G-1-treated OVX UUO model at the transcript level (Fig. 4A). Similarly, CD86-positive cells were significantly reduced in the kidneys of G-1-treated OVX UUO mice than in vehicle-treated OVX UUO mice, as evidenced by IHC staining (Fig. 3B, C). Moreover, renal cortex *Mrc1*, *Il10, Arg-1*, and *Ccl17* transcript levels were significantly reduced in the OVX UUO model with G-1 administration (Fig. 4B). Following G-1 treatment, CD206-positive cells were significantly and consistently decreased (Fig. 3B, C). We also conducted a flow cytometry-based analysis of myeloid cell populations in the kidneys of four groups. Our results revealed a significant decrease in the numbers of CD45+ CD11b+ positive cells in the kidneys of G-1-treated OVX UUO mice compared to the vehicle-treated OVX UUO mice (Fig. 4C, D). There were significantly fewer total macrophages (F4/80high), significantly fewer M1 (F4/80-CD86high) and M2 type (F4/80-CD206high) macrophages (Fig. 4C, D) in G-1-treated OVX UUO mice.

Together, these results suggested that activation of GPER1 significantly downregulated the M1 and M2 macrophage recruitment in the kidney tissues in the OVX UUO model.

### Activation of GPER1 in male mice also attenuates UUO-induced renal fibrosis

**Fig. 5:**
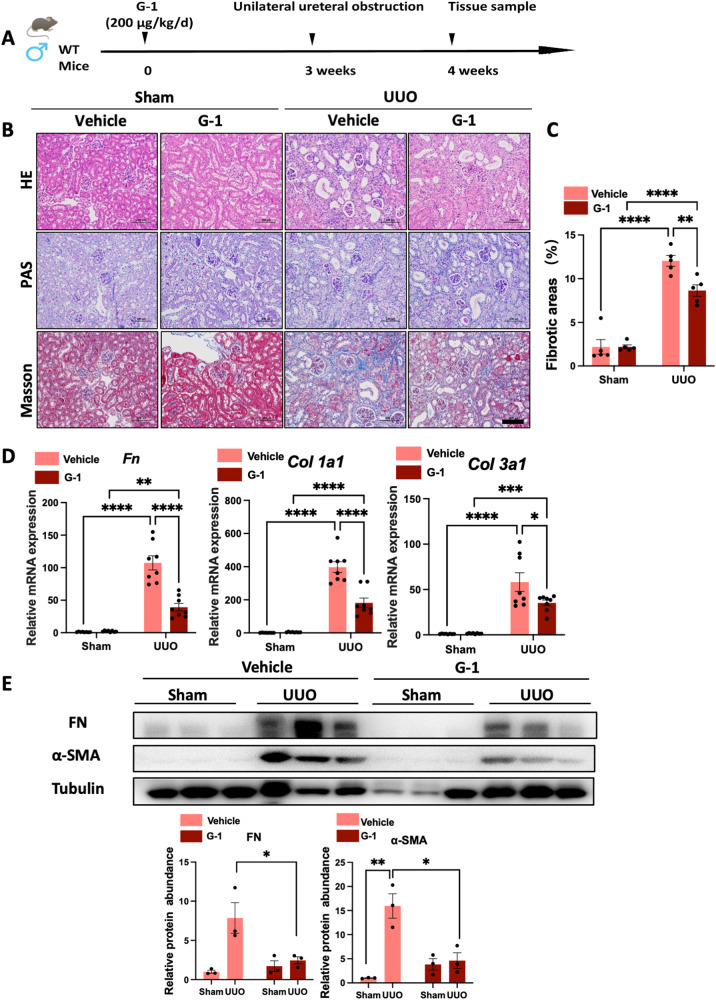
GPER1 activation ameliorates renal fibrosis in the male murine UUO model. **A** Schematic diagram shows the experimental design. **B** Representative images of kidney sections of H&E, Masson’s trichrome, and PAS staining of male mice subjected to UUO for 7 days with or without G-1 treatment. Scale bars: 100 μm. **C** Quantification of Masson’s trichrome staining (*n* = 5 per group). **D** Relative mRNA expression of *Fn, Col1a1*, and *Col3a1* in sham and obstructed kidney samples from vehicle or G-1-treated male mice (*n* = 8 in each group). **E** Representative western blots of Fibronectin and α-SMA in sham and obstructed kidney tissues from male mice treated with or without G-1 (*n* = 3). Densitometry analysis was performed to quantify protein expression. Data are shown as means ± SEM. Statistical analysis by two-way ANOVA with Tukey’s test. *P < 0.05,**P < 0.01, ***P < 0.001, ****P < 0.0001.

To assess whether activating GPER1 yielded similar results in the male UUO mice model, we also performed the UUO surgeries on 8-week-old male mice. The mice were pretreated with the same dose of G-1 by subcutaneous implantation of alzet minipumps 3 weeks before UUO modeling (Fig. 5A). We initially compared the renal fibrotic changes between the vehicle-treated UUO male mice and the G-1-treated UUO male mice (Fig. 5B). Quantification of Masson’s trichrome staining revealed a significant improvement in collagen deposition in G-1-treated UUO male mice (Fig. 5C). Consistent with Masson’s trichrome staining, the mRNA expression of *Col1a1*, *Col3a1*, and *Fn*, as well as protein expression of α-SMA and Fibronectin, were all significantly downregulated after G-1 treatment (Fig. 5D, E).

**Fig. 6:**
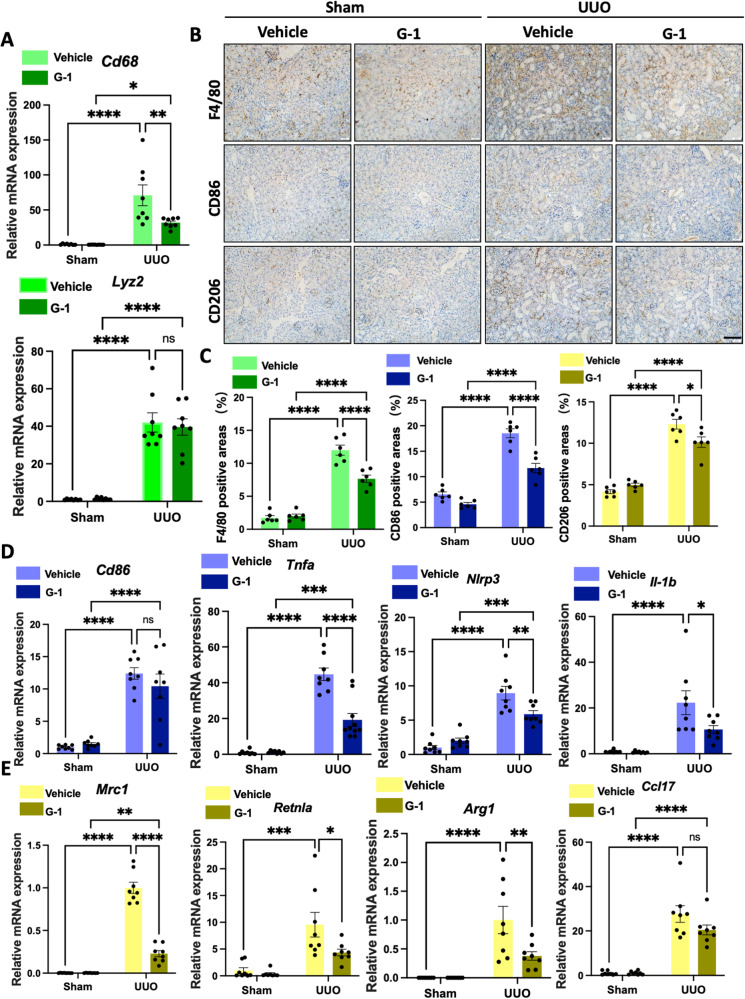
GPER1 activation reduces renal macrophage infiltrations in the male UUO model. **A** Relative mRNA expression of *Cd68* and *Lyz2* in sham and obstructed kidneys from male mice treated with or without G-1 (*n* = 8 in each group). **B** Representative images of macrophage (F4/80), M1 macrophage (CD86), and M2 macrophage (CD206) immunohistochemistry-stained kidney samples from male mice subjected to UUO, treated with or without G-1. Scale bars: 100 μm. **C** Quantification of F4/80, CD86, and CD206 positive stained area in four groups (*n* = 6). **D**, **E** Relative mRNA expression of M1 macrophage-associated proinflammatory genes *Cd86, Tnf-a, Nlrp3*, *Il-1b, Mrc1, Retnla, Arg-1*, and *Ccl17* of the sham or obstructed kidney samples of OVX mice treated with or without G-1. (*n* = 8 in each group). Data are shown as means ± SEM. Two-way ANOVA with Tukey’s test. *P < 0.05,**P < 0.01, ***P < 0.001, ****P < 0.0001.

To explore the effect of G-1 on inflammation and immune response in the male UUO model, we also compared the M1-associated chemokines and proinflammatory cytokines, as well as M2 macrophage-related markers between the vehicle-treated UUO male mice and G-1-treated UUO male mice. There was a significant reduction in *Cd68* (Fig. 6A)*, Nlrp3*, *Il-1b*, and *Tnf-a* (Fig. 6D) mRNA expression in the G-1-treated male UUO mice model compared to the vehicle-treated male UUO mice model. This is consistent with the findings in the OVX UUO model after G-1 treatment. However, *Cd86* mRNA remained unchanged (Fig. 6D), whereas there was a significant reduction in *Mrc1, Retnla, Arg-1*, and *Ccl17* mRNA expressions (Fig. 6E). These results were validated by IHC staining (Fig. 6B, C).

Taken together, these findings confirmed that GPER1 activation protected the male mice against UUO-induced renal fibrosis. However, in contrast to the activation of GPER1 in female mice, which leads to suppression of both M1 and M2 macrophage polarization, GPER1 activation in male mice mainly resulted in the inhibition of M2 macrophage polarization, suggesting sex differences in GPER1 activation in the kidney of UUO mice.

### *Gper1* deletion exacerbates renal fibrosis in male UUO-induced renal fibrosis model

To identify whether *Gper1* deletion affects renal fibrosis progression, *Gper1* global knockout mice were generated by the CRISPR/Cas9 gene edition technique (Supplementary Fig. 1A, B). An approximate 90% decrease in *Gper1* mRNA and protein was observed in the kidney tissue of knockout mice compared to the wild-type littermates (Supplementary Fig. 1C, D). The IHC staining revealed that the GPER1 positive area was primarily distributed in TECs of wild-type mice, whereas there was no GPER1 expression in the *Gper1*-deficient mice (Supplementary Fig. 1E). The *Gper1* gene expression levels in various tissues, including the heart, lung, intestine, and liver, were also downregulated in the *Gper1*^*−/−*^ mice compared with wild-type mice (Supplementary Fig. 1F). Our findings confirmed successful *Gper1* knockout in kidneys and other tissues in *Gper1*^*−/−*^ mice.

**Fig. 7:**
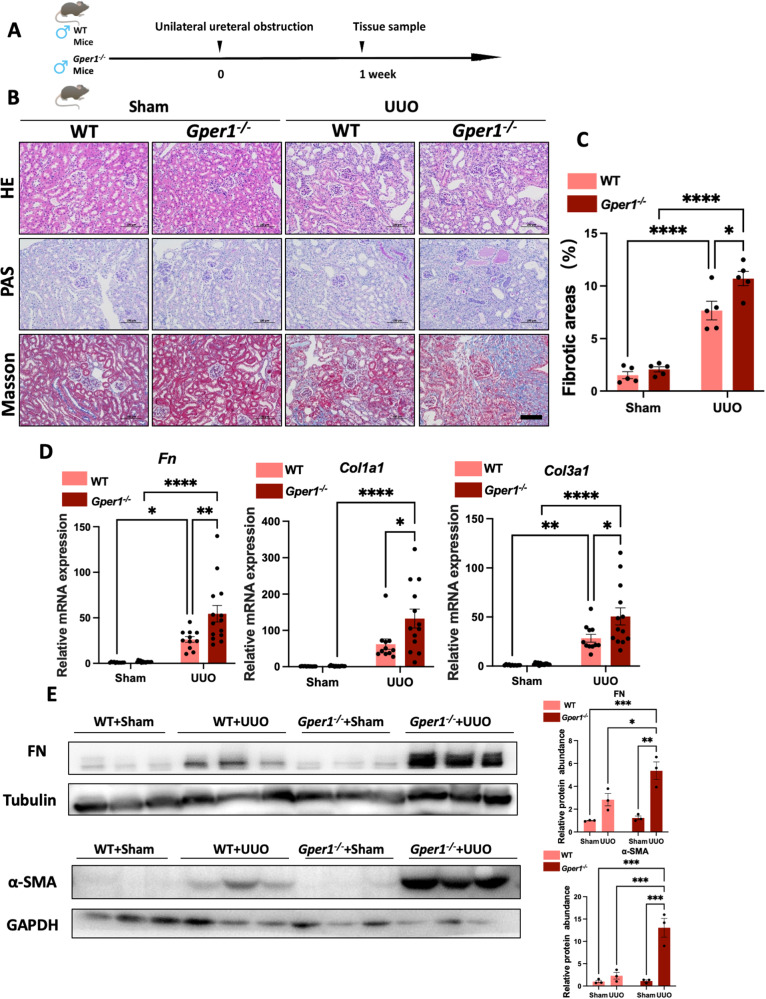
*Gper1* deletion exacerbates renal fibrosis in the UUO model. **A** Schematic diagram shows the experimental design. **B** Representative images of kidney sections of H&E, Masson’s trichrome, and PAS staining of male mice subjected to UUO with or without G-1 treatment for 7 days. Scale bars: 100 μm. **C** Quantification of Masson’s trichrome staining (*n* = 5 per group). **D** Relative mRNA expression for *Fn, Col1a1, Col3a1* of sham and obstructed kidney samples of wild-type or *Gper1*^*−/−*^ male mice (*n* = 11 in wild-type group, *n* = 13 in *Gper1*^*−/−*^ group). **E** Representative western blots of Fibronectin and α-SMA of sham and obstructed kidney tissues from wild-type or *Gper1*^*−/−*^ male mice treated with or without G-1 (*n* = 3). Densitometry analysis was performed to quantify protein expression. Data are shown as means ± SEM. Statistical analysis by two-way ANOVA with Tukey’s test. *P < 0.05,**P < 0.01, ***P < 0.001, ****P < 0.0001.

To evaluate the effect of *Gper1* deficiency on renal fibrosis, the *Gper1* knockout male mice and wild-type male littermates were also subjected to UUO surgeries (Fig. 7A). The PAS, H&E, and Masson’s trichrome staining revealed that *Gper1*-deficient male mice subjected to the UUO model exhibited more collagen synthesis and deteriorated tubular injuries as compared to wild-type mice subjected to UUO (Fig. 7B, C). In UUO murine models, *Gper1* deficiency also accelerated the transcript levels of profibrotic factors, chemokines, and proinflammatory cytokines (Fig. 7D, E, Supplementary Fig. 2A–C). Consistent with G-1-treated male mice, we found that male Gper1-deficient mice subjected to the UUO model exhibited the same reduction in CD68 and CD206 (Supplementary Fig. 2B, D). Nonetheless, CD86 remained unchanged (Supplementary Fig. 2C). These data further confirmed that GPER1 played a critical role in renal fibrosis by *Gper1* deletion. In UUO-induced renal fibrosis, deficiency in *Gper1* also led to enhanced expression of chemokines and proinflammatory cytokines and increased macrophage infiltration.

### GPER1 activation suppresses the immune pathways activation in M0 macrophages

**Fig. 8:**
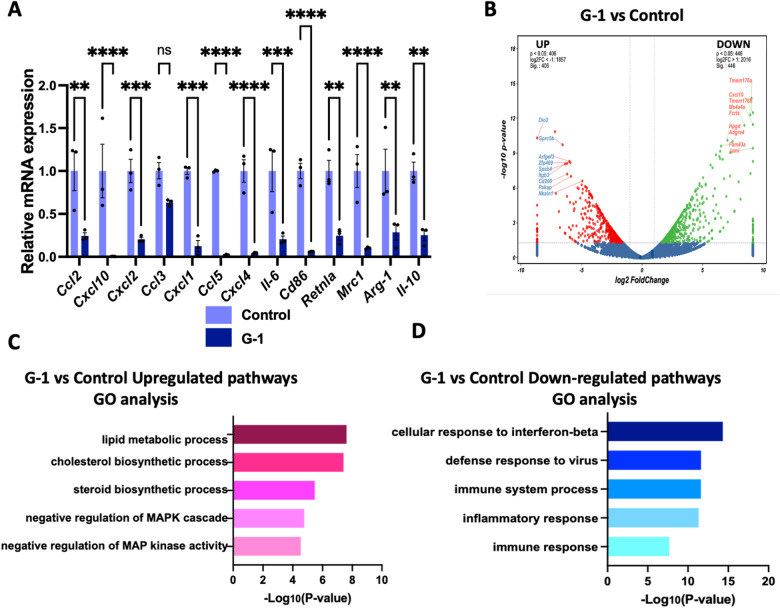
GPER1 activation inhibits macrophage phenotype transition. **A** Relative mRNA expression of M1 and M2 macrophage-associated genes *(Ccl2*, *Cxcl10*, *Cxcl2*, *Ccl3*, *Cxcl1*, *Ccl5*, *Cxcl4*, *Il-6*, *Cd86*, *Retnla*, *Mrc1*, *Arg-1*, *Il-10)* in bone marrow-derived macrophages (BMDMs) from WT mice treated with or without G-1 (*n* = 3). **B** Volcano plot of upregulated and downregulated differentially expressed genes identified between M0 macrophages treated with or without G-1.The log2 FC indicates the mean expression level for each gene. Each dot represents one gene. **C** Gene Ontology analysis of downregulated and upregulated pathways in M0 macrophages with or without G-1 treatment. Data are shown as means ± SEM. Unpaired *t* test. *P < 0.05, **P < 0.01, ***P < 0.001, ****P < 0.0001.

Monocytes derived from bone marrow myeloid progenitors are recognized as the main source of infiltrating macrophages in kidney disease [20]. To evaluate the specific roles of GPER1 in macrophages, BMDMs were isolated and differentiated into M0 macrophages after seven days. First, we tested whether GPER1 activation on macrophages could influence its activation. It was found that G-1 could directly inhibit the polarization of macrophages to both M1 and M2 phenotypes, characterized by downregulation of *Ccl2*, *Cxcl10*, *Cxcl2*, *Cxcl1*, *Ccl5*, *Cxcl4*, *Il-6*, *Cd86, Retnla*, *Mrc1*, *Arg-1*, and *Il-10* (Fig. 8A). To establish the following possible pathways regulated by G-1 in M0 macrophages, we then performed transcriptomic profiling on M0 macrophages with or without G-1 treatment. Heatmap demonstrated the upregulation of 446 genes and downregulation of 406 genes in the G-1-treated versus non-treated BMDMs (Fig. 8B). In addition, GO analysis of the differentially expressed genes revealed upregulation of negative regulation of MAPK cascade and MAP kinase activity, and downregulated cellular response to interferon-beta, defense response to the virus, immune system process, inflammatory response, and immune response (Fig. 8C, D). These results suggested that GPER1 activation inhibited the transition of macrophage phenotype toward inflammatory and profibrotic macrophage.

### GPER1 activation inhibits M0 to M1 macrophages polarization and protects co-cultured TECs from injuries and immune response

**Fig. 9:**
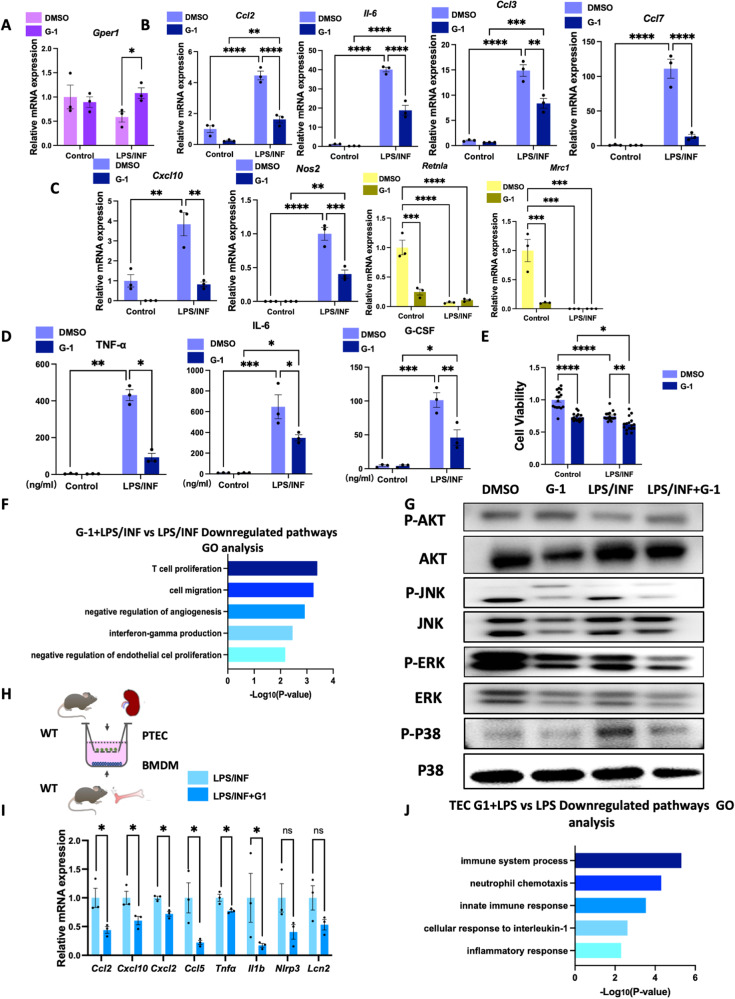
GPER1 regulates inflammatory crosstalk between macrophages and TECs by inhibiting M1 macrophage polarization. **A**–**C** Relative mRNA expression of *Gper1*, proinflammatory cytokines and chemokines (*Ccl2*, *Il-6, Ccl3*, *Ccl7*, *Cxcl10*, *Nos2*), and M2 macrophage markers *Retnla* and *Mrc1* of macrophages in response to LPS/INF with or without G-1 treatment (*n* = 3 in each group). **D** TNF-α, IL-6, and G-CSF expression in the supernatants of macrophages in response to LPS/INF with or without G-1 treatment (*n* = 3 in each group). **E** Cell viability of BMDMs in response to LPS/INF with or without G-1 treatment (*n* = 16 in each group). **F** Gene Ontology analysis of downregulated pathways in macrophages stimulated with LPS/INF with or without G-1 treatment. **G** Representative western blots of the protein levels of phosphorylation of AKT, MAPK/JNK, MAPK/ERK, and MAPK/P38 of BMDMs in response to LPS/INF with or without G-1 treatment. **H** Schematic representation of co-culture experiment with primary BMDMs in the lower chamber and primary PTECs in the upper chamber using a trans-well system. **I** Relative mRNA expression of *Ccl2*, *Cxcl10*, *Cxcl2*, *Ccl5*, *Tnf-a, Il-1b*, *Nlrp3* and *Lcn2* in PTECs co-cultured with M1 macrophages pretreated with or without G-1 (*n* = 3). **J** Gene Ontology analysis of downregulated pathways of PTECs co-cultured with M1 macrophages pretreated with or without G-1. Values are given as mean ± SEMs. Two-way ANOVA with Tukey’s test. *P < 0.05,**P < 0.01, ***P < 0.001, ****P < 0.0001.

Then, in response to LPS/INFγ(INF) stimulation with or without G-1 treatment, we focused on M0 macrophages polarized into M1 macrophages. Compared to non-stimulated macrophages, increased expression of chemokines and proinflammatory cytokines (*Ccl2*, *Il-6, Ccl3*, *Ccl7*, *Cxcl10*, *Nos2*) were observed in macrophages in response to LPS and INF (Fig. 9B, C). In comparison, the upregulated expression of chemokines and proinflammatory cytokines induced by LPS and INF was significantly suppressed by G-1 (Fig. 9B, C). The M2 macrophage markers *Mrc1* and *Retnla* were significantly downregulated in both BMDMs in response to LPS and INF treated with or without G-1 (Fig. 9C). We also confirmed that after LPS/INF stimulation, TNF-α, IL-6, and G-CSF were significantly elevated in the supernatants of M1 macrophages (Fig. 9D). The G-1 treatment downregulated TNF-α, IL-6, and G-CSF from the supernatants of M1 macrophages (Fig. 9D). Consistent with the in vivo study, using a CCK8 kit, G-1 treatment significantly downregulated macrophage proliferation in both groups with or without LPS/INF stimulation (Fig. 9E).

Next, we performed GO enrichment analysis on the DEGs from M1-polarized macrophages with or without G-1. The downregulated genes were mainly enriched in pathways that affect T-cell proliferation, cell migration, negative regulation of angiogenesis, and interferon-gamma production (Fig. 9F). Consistent with RNA sequencing, immunoblotting further validated that G-1 treated BMDMs with or without LPS/INF stimulation exhibited downregulated levels of phosphorylation of MAPK/JNK, MAPK/ERK, and MAPK/P38 compared with BMDMs without G-1 treatment (Fig. 9G).

It has been reported that the recruitment of M1 macrophage could cause severe injuries to TECs, further activating the immune response and aggravating renal damage [21]. To investigate how GPER1 activation in macrophages could contribute to reduced tubular injuries in the UUO model, we established an in vitro model via co-culture of BMDMs with TECs using a trans-well assay (Fig. 9H). Based on qPCR results, *Ccl2*, *Cxcl10*, *Cxcl2*, *Ccl5*, *Tnf-a*, and *Il-1b* mRNA expression of TECs were significantly restrained when co-cultured with M1 macrophages pretreated with G-1 (Fig. 9I). A decreasing trend of *Nlrp3* and *Lcn2* mRNA was also observed (Fig. 9I), indicating that GPER1 activation in macrophages could protect co-cultured TECs from injuries. We also used RNA sequencing to identify the enriched pathways of differentially expressed genes. The result revealed that G-1 pretreatment in M1 macrophages induced downregulation of pathways enriched in the immune system process, neutrophil chemotaxis, innate immune response, and cellular response to interleukin-1 and inflammatory response in the co-cultured TECs (Fig. 9J).

Altogether, these data revealed that GPER1 activation significantly reduced the expression of inflammatory factors by inactivating MAPK pathways in BMDMs, hence protecting the co-cultured TECs against injuries and immune response. These findings highlight the importance of GPER1 in macrophages during the progression of renal fibrosis. Specifically, GPER1 is crucial in preventing TECs from developing into immune-like cells, exacerbating kidney inflammation, and contributing to End-Stage Renal Disease (ESRD) development.

### GPER1 deficiency accelerates LPS/INF-induced inflammatory pathways in macrophages and enhances tubular epithelial injuries

To explore whether loss of GPER1 accelerates inflammatory pathways in macrophages, we also isolated BMDMs from WT and *Gper1* knockout mice. The level of *Gper1* mRNA was significantly downregulated in *Gper1*^*-/-*^ BMDMs (Supplementary Fig. 4A). Increases in *Cxcl10*, *Cxcl2*, *Cxcl1*, *Ccl7*, and *Ccl4* mRNA were seen in WT BMDMs after LPS/INF treatment, whereas the *Gper1* ablation further enhanced the expression of the inflammatory cytokines (Supplementary Fig. 4B). Additionally, loss of *Gper1* in M1 macrophages caused increased injuries in co-cultured TECs (Supplementary Fig. 4C), as demonstrated by increased *Nlrp3* and *Ccl7* mRNA levels and an increasing trend in *Lcn2* and *Il1b* mRNA (Supplementary Fig. 4D). These results confirmed that *Gper1* deletion enhanced macrophages’ response to LPS/INF, leading to increased impairment in co-cultured TECs.

### Profibrotic factors are downregulated in kidney fibroblast co-cultured with G-1-pretreated M2 macrophages

**Fig. 10:**
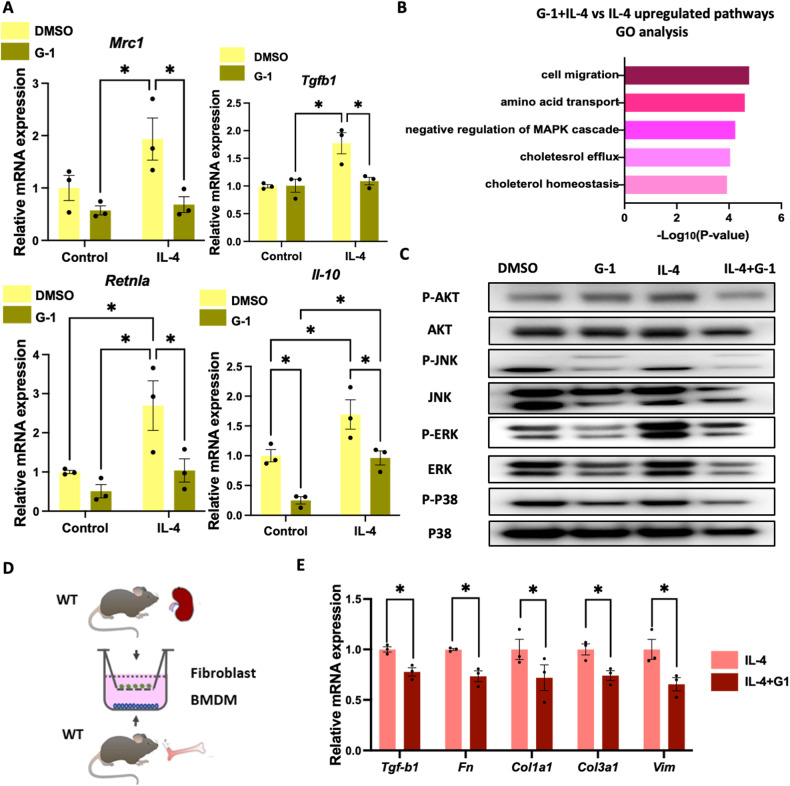
Activation of GPER1 decreases profibrotic factors by inhibiting M2 macrophage polarization. **A** Relative mRNA expression of *Mrc1*, *Tgfb1, Retnla*, and *Il10* in macrophages in response to IL-4 with or without G-1 treatment (*n* = 3). **B** Gene Ontology analysis of upregulated pathways in macrophages stimulated with IL-4 with or without G-1 treatment. **C** Representative western blots of the protein levels of phosphorylation of AKT, MAPK/JNK, MAPK/ERK, and MAPK/P38 pathways in macrophages in response to IL-4 treated with or without G-1. **D** Schematic representation of co-culture experiment with BMDMs in the lower chamber and primary renal fibroblasts in the upper chamber. **E** Relative mRNA expression of *Tgfb1*, *Fn*, *Col1a1, Col3a1*, and *Vim* in primary renal fibroblast co-cultured with M2 macrophages pretreated with or without G-1 (*n* = 3). Data are shown as means ± SEM. Two-way ANOVA with Tukey’s test. *P < 0.05,**P < 0.01, ***P < 0.001, ****P < 0.0001.

Few studies have been conducted on the role of GPER1 in M2 macrophage. To explore whether GPER1 activation impacted the macrophage phenotype in response to IL-4, BMDMs were extracted and polarized into M2 macrophage by IL-4 with or without G-1 treatment. As shown in Fig. 10A, *Mrc1*, *Tgfb1, Retnla*, and *Il10*, and mRNA expression was significantly increased after IL-4 treatment, whereas G-1 treatment prevented IL-4-induced M0 to M2 macrophage polarization with a significant reduction in the *Mrc1*, *Retnla*, *Il-10*, and *Tgfb1* mRNA. The transcriptomic analysis further revealed that G-1 activation upregulated pathways enriched in amino acid transmembrane transport, negative regulation of MAPK cascade, cholesterol homeostasis, inactivation of MAPK activity, and cholesterol efflux (Fig. 10B). Immunoblotting also validated that G-1 reduced the activation of MAPKs in macrophages’ response to IL-4 (Fig. 10C). These findings confirmed that GPER1 activation also affects BMDMs polarized into M2 phenotype, mainly through the negative regulation of MAPK cascade, which is also involved with cholesterol efflux and homeostasis.

In addition to their anti-inflammatory roles, M2 macrophages have recently been recognized to take an essential profibrotic role in tissue fibrosis. They are known to release large amounts of profibrotic factors such as TGF-β1 [22]. To evaluate whether GPER1 activation in M2 macrophage ameliorates the transition of fibroblast into myofibroblast, we co-cultured M2 macrophages pretreated with or without G-1 with primary kidney fibroblasts (Fig. 10D). The M2 macrophages pretreated with G-1 effectively decreased the expression of profibrotic factors of the co-cultured fibroblast, including *Tgfb1*, *Fn, Col1a1*, *Col3a1*, and *Vim* (Fig. 10E).

**Fig. 11:**
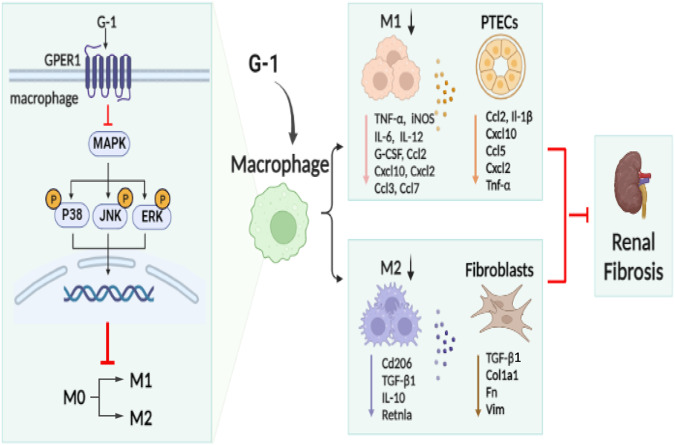
The proposed mechanism mediating the protective action of GPER1 against UUO-induced renal fibrosis. GPER1 agonist G-1 ameliorated UUO-induced renal fibrosis, possibly by inhibiting M0 to M1 and M0 to M2 macrophage polarization and attenuating immune response and fibrosis.

Altogether, these results suggested that G-1 exerted an anti-inflammatory effect on macrophages, and it also prevented the polarization of M0 macrophages into M2 macrophages as well as disrupted fibroblast transition into myofibroblast (Fig. 11).

## Discussion

Recently, several clinical observation studies reveal that male CKD patients present faster progression into ESRD than female CKD patients [23], and women experience lower rates of kidney failure(defined as the need for dialysis or a kidney transplant) compared to men [24]. An IgAN patients retrospective study also implied that the male gender identified as an independent risk factor for poor kidney outcomes [25]. The sex disparities revealed by CKD suggest a role for sex hormones, specifically, a protective role for estrogen and its receptors.

Further studies have investigated the possible mechanisms of gender disparity in renal fibrosis. A study by Kim et al. [26] demonstrated that tamoxifen ameliorated renal tubulointerstitial fibrosis by modulation of estrogen receptor α-mediated transforming growth factor-β1/Smad signaling pathway. Furthermore, Cao et al. [27] confirmed that activation of estrogen receptor β attenuated renal fibrosis by suppressing the transcriptional activity of smad3. These studies indicated estrogen receptors and modulators played significant roles in retarding renal fibrosis progression.

To better understand how estradiol saves the kidney from injuries, our study first identified GPER1, the de novo estrogen receptor’s role in protecting against renal fibrosis. Through in vivo and in vitro studies, we found that activation of GPER1 could attenuate M1 and M2 macrophage polarization. Knockout of *Gper1* also confirmed that GPER1 was highly involved in macrophage activation and recruitment. Similarly, our study and others showed that GPER1 activation attenuated LPS/INF-induced M1 macrophage polarization in mouse macrophage cell lines, peritoneal macrophages, and primary cultured murine BMDMs [9, 10, 28]. The GPER1 depletion further enhanced the macrophage’s response to LPS/INFγ, consistent with a previous study [10]. Equally, our results corroborate other studies in kidney-diseased areas, showing that inhibition of macrophage recruitment ameliorated renal fibrosis [29, 30]. In contrast to early studies indicating that GPER1 activation was only involved in M1 macrophage polarization derived from multiple studies, our data suggested that activating GPER1 inhibited M2 macrophage polarization, leading to ameliorated renal fibrosis. These results agree with recent findings by Feng et al. [31] that stimulating macrophage M2 polarization enhances renal fibrosis. These findings provide insights into the cellular and molecular mechanisms involved in M2 macrophage-mediated renal fibrosis.

Previous studies reported that SerpinB2 played crucial roles in macrophage polarization and tubule-macrophage crosstalk [32]. We further investigated the interactions between M1-polarized macrophages and TECs and M2-polarized macrophages and fibroblasts. We found that activating GPER1 in M1 macrophages impaired inflammatory signaling, thereby protecting TECs from immune activation and injuries. Moreover, activating GPER1 in M2 macrophages suppressed the transition of resident fibroblast to myofibroblast. These interactions highlight the diverse roles of GPER1 in regulating the two different macrophage phenotypes and their subsequent crosstalk with renal innate cells.

Using RNA sequencing techniques, we discovered that MAPK inactivation was the most significantly enriched pathway in the DEG sets between macrophages with or without G-1 administration. The immunoblotting results validated this finding. The effects of GPER1 activation on certain pathways have been studied extensively. However, the results are still controversial. The GPER1 activation has been reported to activate protein kinase pathways, including MAPK, and activate PI3K/Akt pathways [33, 34]. Estrogen increased phosphorylated MAPK in fibroid cells but not in myometrial cells [35], indicating different subsequent pathway activations in various cell types and tissues. On the other hand, G-1 generally reduced ERK1/2 phosphorylation in various types of smooth muscle cells [36, 37]. These findings are consistent with progestin-induced ERK inactivation via GPER1 [38]. Filardo et al. [39] explored the possible upstream signaling factors that led to ERK1/2 inhibition. They found that GPER1 activation suppressed EGF-induced ERK1/2 activation by stimulating adenylyl cyclase activity in SBRK3 cells. In this study, the inactivation of MAPK pathways in response to GPER1 activation in macrophages supported the aforementioned findings. However, further studies are needed to identify the precise mechanisms underlying the differential effects of GPER1 activation on the MAPK pathway.

Additionally, GPER1 agonist G-1 is protective against inflammation through macrophage modulation in various neuro-diseases, including multiple sclerosis [40], Parkinson’s disease [41, 42], and traumatic brain injury [43]. A recent publication in Science revealed that G-1 might preserve fetal health via INF signaling regulation in maternal tissues [44]. A study by G. Sharma et al. [45] revealed that GPER1 selective agonism is a potential therapeutic approach for diabetes and associated metabolic abnormalities. This study established a new role for GPER1 agonist G-1 in counteracting renal fibrosis, demonstrating its versatility and therapeutic ability in treating various diseases.

Interestingly, we also observed gender disparities in this work in response to G-1-mediated-GPER1 activation. Both M1 and M2 macrophage markers were shown to be downregulated in response to G-1 therapy in female OVX UUO mice; however, only M2 macrophage markers CD206 were found to be significantly downregulated in UUO male mice. Despite M1 macrophages being unaltered, male UUO mice showed noticeably decreased levels of inflammatory cytokines and chemokines. A recent study by Wu et al. [46], using proximal tubule translational profiling during kidney fibrosis, also showed sex dimorphism on proinflammatory and long noncoding RNA expression patterns in tubules, indicating gender disparities in different cell types. Another study by McCrimmon et al. also observed striking sex differences in tubular injuries. The results showed that male mice shut down fatty acid oxidation and several other metabolism-related pathways; female mice had a significantly weaker transcriptional response in metabolism, but activation of inflammatory pathways was prominent [47]. Interestingly, it was also verified that male macrophages had higher expression of cell surface TLR4 and responded to LPS, a TLR4 ligand, with a higher production of both IL-1β and CXCL10 and with a lower production of the prostaglandin PGE(2) than female-derived macrophages [48]. However, sex differences in GPER1 distribution between males and females were observed in some studies but not in others [49]. Furthermore, another study indicated dehydroepiandrosterone could activate GPER1 to mediate cell death in Nig-stimulated inflammatory macrophages [50]. Testosterone is also immunosuppressive, as it inhibits B cell lymphopoiesis in the bone marrow [51]. Therefore, understanding the roles of sex hormones in activating GPER1 is imperative in future studies.

The study has the following limitations: first, this study only focused on the macrophage’s oversimplified M1 and M2 phenotypes. However, single-cell RNA-sequencing (scRNA-seq) has recently revealed the immense complexity of macrophage populations in the development and progression of CKD [52]. Also, using human liver and lung scRNA-Seq, Fabre et al. identified a subset of CD9+ TREM2+ macrophages with profibrotic roles in fibrotic tissues [53]. To precisely analyze the subsets of the macrophages and the roles played by GPER1 activation, it would be necessary to perform single-cell RNA sequencing on UUO models treated with or without GPER1 agonist. In addition, female mouse macrophages should be used to unveil the GPER1’s role in the sexual dimorphism in renal fibrosis progression. Moreover, studies with more specific Cre recombinase models for macrophages and tubule epithelial cells are desirable to clarify the specific roles of GPER1 in different cell types that contribute to the progression of renal fibrosis. Further studies are required to provide a definitive answer on how GPER1 activates the following pathways.

In conclusion, the current study found that GPER1 plays an essential role in the control of macrophage inflammatory and profibrotic responses in renal fibrosis. These findings provide a full explanation of how GPER1 influences macrophage-mediated renal fibrosis. Moreover, the study identified G-1 as a potential treatment in renal fibrosis prevention.

### Supplementary information

Supplemental

Original Data File-WB

Original Data File-qPCR

## Data Availability

The data supporting the findings of this study are available from the corresponding author upon reasonable request.
