# Supplementary material for: Activation of GPER1 in macrophages ameliorates UUO-induced renal fibrosis
Source: Cell Death Dis. 2023 Dec 12;14(12):818. doi: 10.1038/s41419-023-06338-2 (PMC10716282; doi:10.1038/s41419-023-06338-2)
Supplement: Supplementary file 1 — Supplemental [file 41419_2023_6338_MOESM1_ESM.docx]

**Supplementary Materials**

Fig S1. Validation of *Gper1* knockdown in *Gper1^-/-^* mice.

Fig S2. Deletion of *Gper1* mitigated macrophage infiltration and inflammation.

Fig S3. Differentially expressed genes in BMDMs in response to LPS/INF treated with or without G-1.

Fig S4. *Gper1* deletion aggravates inflammation of macrophages and enhances epithelial cell damage.

Table S1. Primers for CRISPR/Cas9 edited *Gper1^-/-^* mice.

Table S2. Sequences of oligonucleotide primers used for qPCR.

Table S3. List of primary antibodies.

Table S4. Antibodies used for flow cytometry analysis.

**
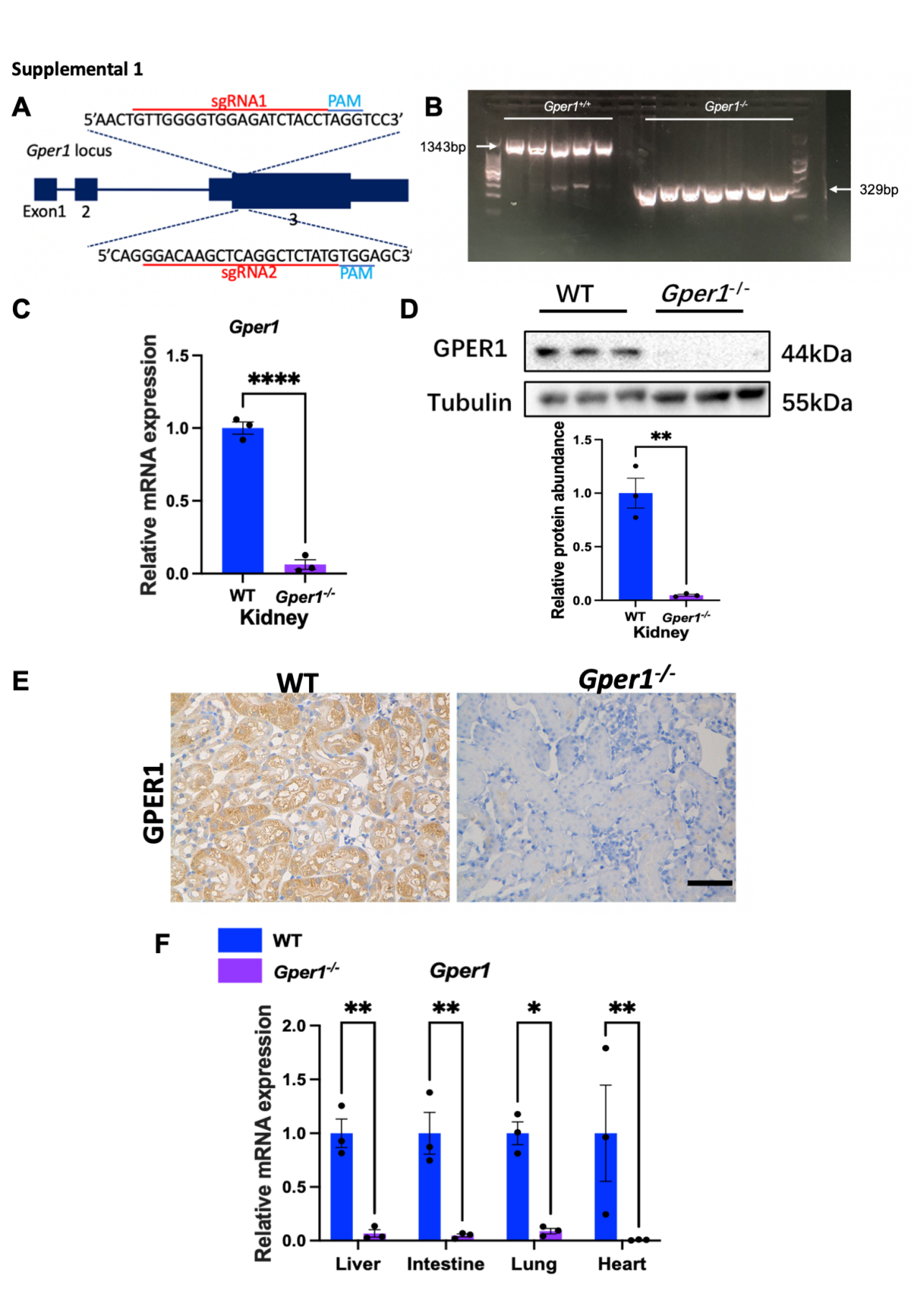
**

**Supplementary figure 1 Confirmation of *Gper1* knockdown in *Gper1^-/-^* mice**

**(A)**Schematic of CRISPR/Cas9 mediated *Gper1* deletion.

**(B)**Genotyping of wild-type mice and *Gper1^-/^*^-^ mice

**(C)**Relative *Gper1* mRNA expression levels of the kidney samples from wild-type mice and *Gper1^-/^*^-^ mice.

**(D)**Representative Western blots of the protein levels of kidney tissues from wild-type or *Gper1^-/-^* mice (n=3). Densitometry analysis was performed to quantify protein expression.

**(E)**Representative images of GPER1 immunohistochemistry staining of wild-type and *Gper1^-/^*^-^ mice.

**(F)**Relative *Gper1* mRNA expression levels of the liver, intestine, lung and heart from wild-type mice and *Gper1^-/^*^-^ mice. Data are shown as means± SEM. Statistical analysis by unpaired t test. *P＜0.05,**P＜0.01, ***P＜0.001, ****P＜0.0001.

**
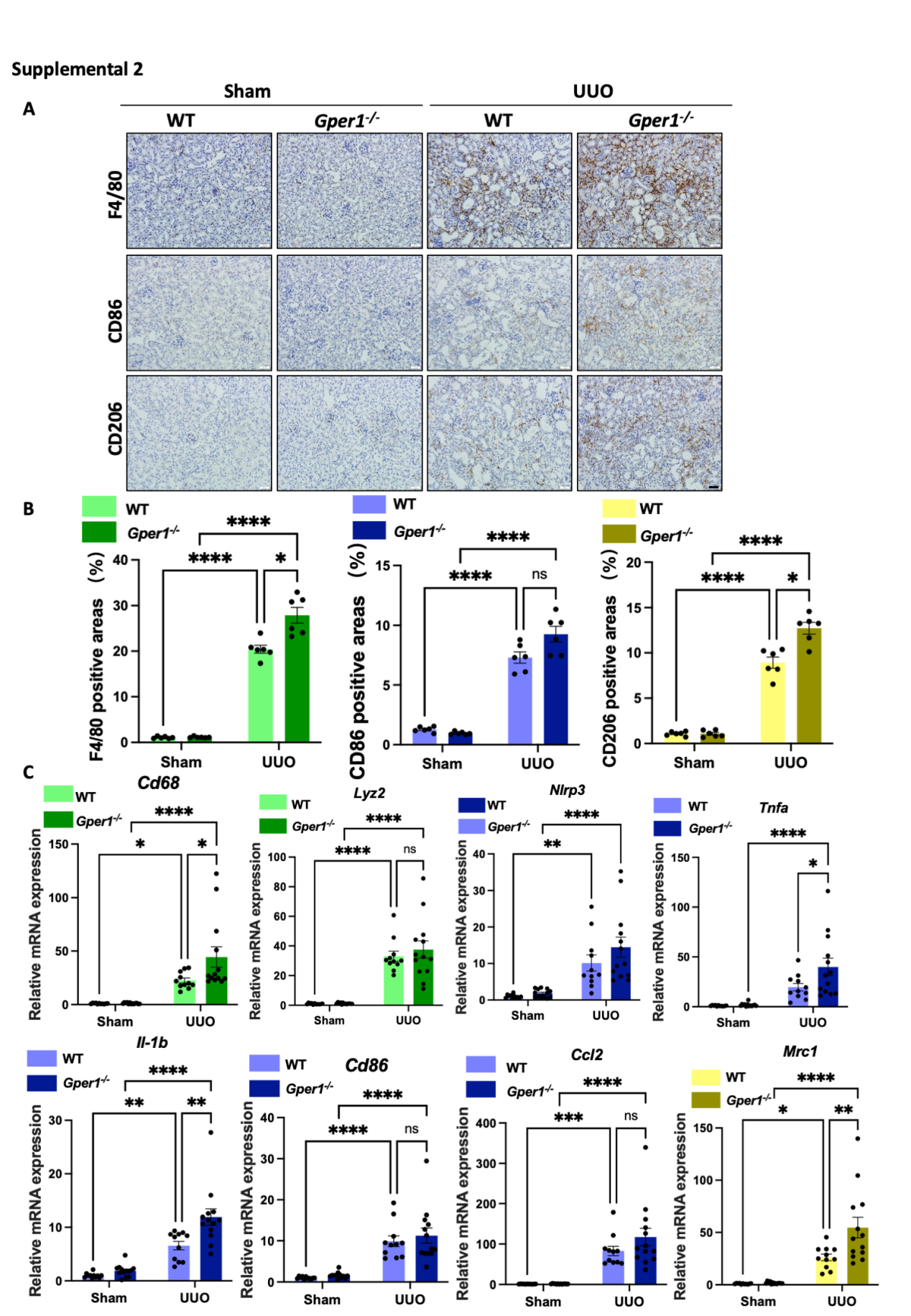
**

**Supplementary figure2 Deletion of *Gper1* mitigated macrophages infiltration and inflammation**

**(A)** Representative macrophage (F4/80), M1 macrophage (CD86) and M2 macrophage (CD206) IHC image in kidney sections of mice treated as described above.

**(B)** Quantification of F4/80, CD86 and CD206 positive stained area in four groups (n=6).

**(C)** Quantitative RT-PCR of mRNA for M1 macrophage-associated proinflammatory genes *(Cd68, Lyz2, Nlrp3, Tnf-α*, *Il-1b*, *Cd86,*  *Ccl2)* and M2 macrophage-associated genes (*Mrc1)* in the kidneys of mice treated as described above(n=11 in wild-type group, n=13 in *Gper1^-/-^* group). Original magnification,×200. Scale bar: 50μm. Values are given as mean ± SEMs. Two-way ANOVAs with Bonferroni multiple comparison test. **P*＜0.05,***P*＜0.01, ****P*＜0.001, *****P*＜0.0001.

**
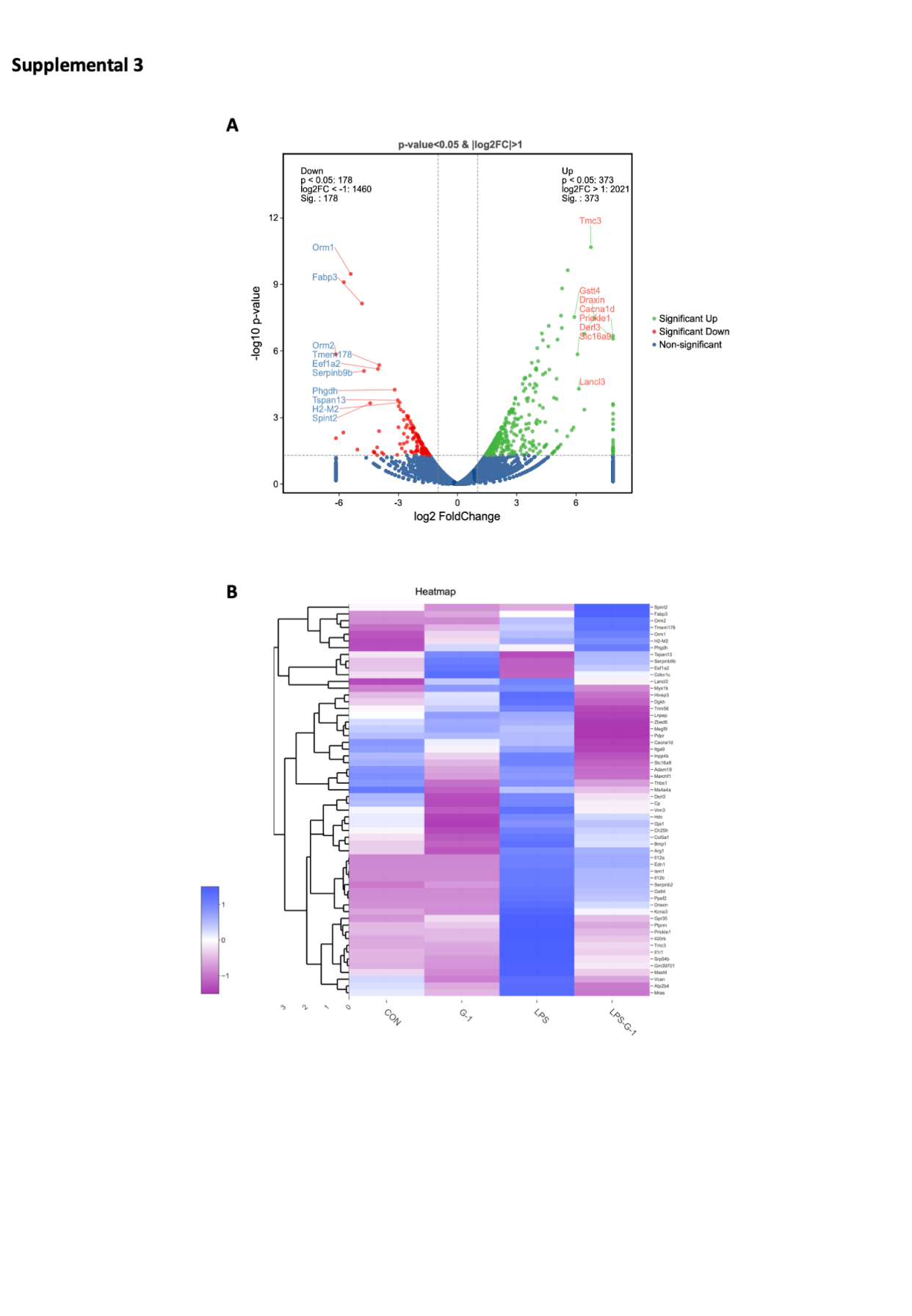
**

**Supplementary figure3 Differentially expressed genes in BMDMs in response to LPS/INF treated with or without G-1**

1. Volcano plot of both upregulated and downregulated differentially expressed genes identified between the LPS/INF treated BMDMs and G1+ LPS/INF treated BMDMs. The log2 FC indicates the mean expression level for each gene. Each dot represents one gene.
2. Heatmaps of genes identified in BMDMs in response to LPS/IFN with or without G-1 treatment. Gene intensities were log2 transformed and are displayed as colours ranging from pink to blue. Rows are clustered using correlation distance and average linkage.

**
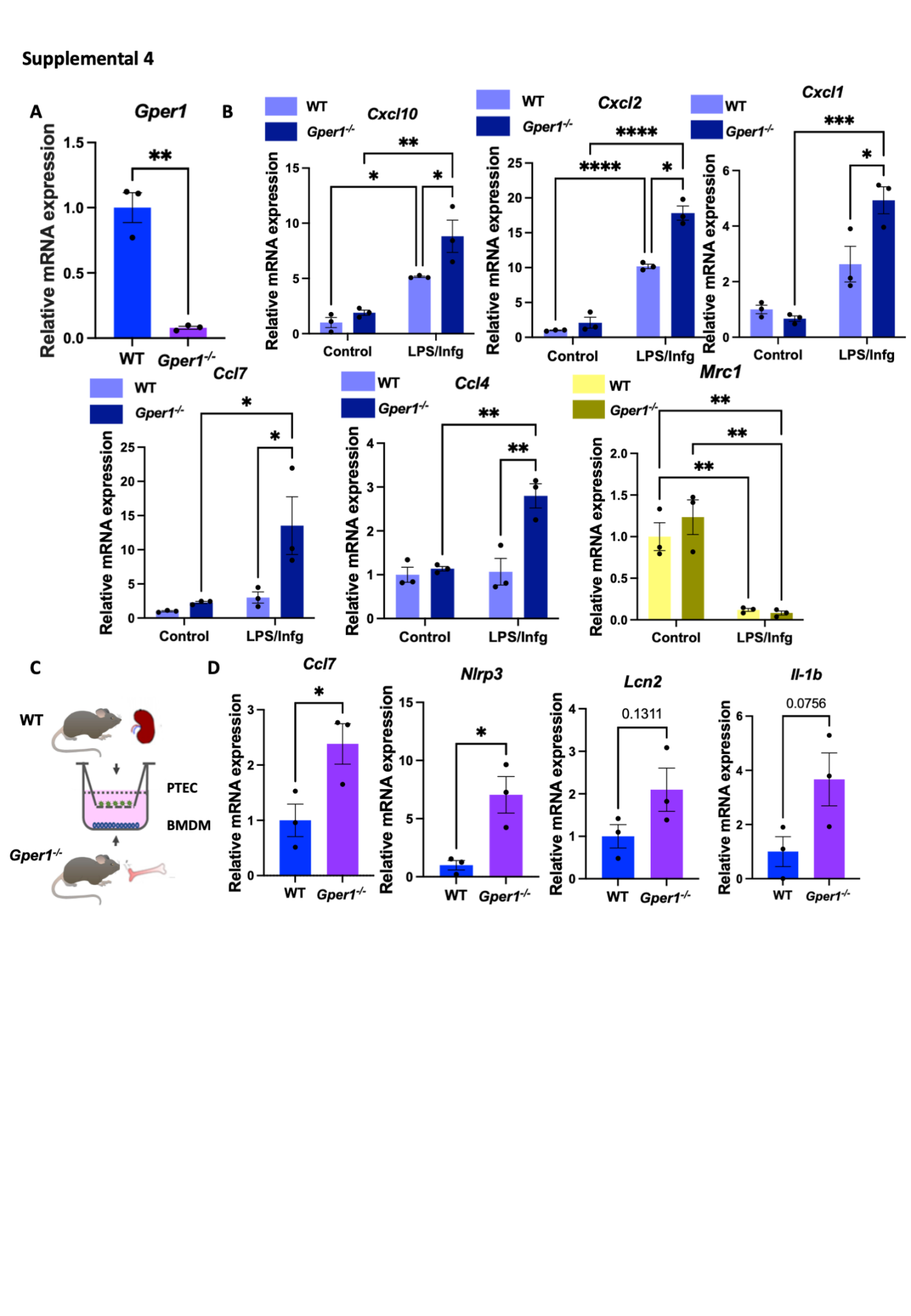
**

**Supplementary figure 4 *Gper1* deletion aggravates [inflammation](javascript:;) of macrophages and enhances epithelial cells damage.**

**(A)**Relative *Gper1* mRNA expression levels from BMDMs from wild-type and *Gper1^-/^*^-^ mice (n=3).

**(B)**Quantitative RT-PCR of mRNA for proinflammatory cytokines (*Cxcl10*, *Cxcl2*, *Cxcl1*, *Ccl4*, *Ccl7 and Mrc1*) in M1 macrophages from WT and *Gper1^-/-^* mice (n=3).

**(C)** Schematic diagram showed the experimental design. Schematic representation of coculture experiment with BMDMs (from WT and *Gper1^-/-^* mice) in the lower chamber and PTECs in the upper chamber.

**(D)**Quantitative RT-PCR of mRNA for *Nlrp3*, *Ccl7*, *Lcn2,* and *Il-1b* in PTECs co-cultured with M1 macrophages from WT and *Gper1^-/-^* mice (n=3). Data are shown as means ± SEM. Two-way ANOVAs with Tukey’s test. **P*＜0.05,***P*＜0.01, ****P*＜0.001, *****P*＜0.0001.

**Supplementary Table 1. Primers for CRISPR/Cas9 edited *Gper1* KO mice**

| **Primer** | **Sequence** |
| --- | --- |
| Gper1-sgRNA1 | GTTGGGGTGGAGATCTACCTAGG |
| Gper1-sgRNA2 | GGACAAGCTCAGGCTCTATGTGG |
| Gper1-PCR-F | ACTAACAGGCTCCCAGGACGAT |
| Gper1-PCR-R | TGACATAGCTTAGCCCACTCAC |

**Supplementary Table 2. Sequences of oligonucleotide primers used for qPCR**

| **Gene** | **Primer Forward** | **Primer Reverse** |
| --- | --- | --- |
| *Fn* | ATGGTACAGCTGATCCTGCC | GCCCTGGTTTGTACCTGCTA |
| *Col3a1* | ACAGCTGGTGAACCTGG | ACCAGGAGATCCATCTCGAC |
| *Col1a1* | CCCAGCCGCAAAGAGTCTAC | AGCATACCTCGGGTTTCCAC |
| *Tgf-b1* | GGGAAGCAGTGCCCGAACCC | TGGGGGTCAGCAGCCGGTTA |
| *Nlrp3* | AGCCTTCCAGGATCCTCTTC | CTTGGGCAGTTTCTTTC |
| *Il-1b* | TGCCACCTTTTGACAGTGATG | TGATGTGCTGCTGCGAGATT |
| *Tnfa* | ATGGGCTCCCTCTCATCAGT | GCTTGGTGGTTTGCTACGAC |
| *Il-6* | GTTCTCTGGGAAATCGTGGA | TGTACTCCAGGTAGCTATGG |
| *Nos2* | GTTCTCAGCCCAACAATACAAGA | GTGGACGGGTCGATGTCAC |
| *Cd68* | AAGGTCCAGGGAGGTTGTGA | GGCTCTGATGTAGGTCCTGT |
| *Lyz2* | AAGGCATTCGAGCATGGGTG | TCGAGGGAATGTGACCTCTCT |
| *Cd86* | GATGGACCCCAGATGCACCAT | ACCAGCTCACTCAGGCTTATG |
| *Ccl2* | CAGCCCAGCACCAGCAC | GCGTTAACTGCATCTGGCTG |
| *Cd206* | TCTTTGCCTTTCCCAGTCTCC | TGACACCCAGCGGAATTTC |
| *Il-10* | GCTATGCTGCCTGCTCTTACT | CCTGCTGATCCTCATGCCA |
| *Arg1* | CAGAAGAATGGAAGAGTCAG | CAGATATGCAGGGAGTCACC |
| *Retnla* | CCCTGCTGGGATGACTGCTA | TCCACTCTGGATCTCCCAAGA |
| *Ccl17* | ACCTTCACCTCAGCTTTTGGT | CTGGAACACTCCACTGAGGTC |
| *Cxcl10* | CCACGTGTTGAGATCATTGCC | GAGGCTCTCTGCTGTCCATC |
| *Cxcl2* | CCCAGACAGAAGTCATAGCCAC | TGGTTCTTCCGTTGAGGGAC |
| *Ccl3* | AAACTTGCAAAACCTGAGAAGC | GCCATTCTACTTGTCTCTGGTGA |
| *Ccl7* | ACCTTCACCTCAGCTTTTGGT | CTGGAACACTCCACTGAGGTC |
| *Gper1* | CTTCTGTTCCTCTCCTGC | CTTCTGATTTTCTGTTGG |
| *Ccl5* | CCCTACAAGAGACTCTGGCTC | TACCAGGGAGTAGAGTGGGG |
| *Lcn2* | CCGACACTGACTACGACCAG | CATTGGTCGGTGGGAACAGA |
| *Cxcl1* | TGGTTCTTCCGTTGAGGGAC | TGTTGTCAGAAGCCAGCGTT |
| *Ccl4* | CTTCTGTGCTCCAGGGTTCTC | TCTTTTGGTCAGGAATACCACAGC |
| *Vim* | GGATCAGCTCACCAACGACA | GGTCAAGACGTGCCAGAGAA |

**Supplementary Table 3. List of primary antibodies**

| **Primary Antibodies** | **Catalog No.** | **Dilution** | **Company** |
| --- | --- | --- | --- |
| p-AKT | 4060T | 1:1000 | CST |
| AKT | 4691T | 1:1000 | CST |
| p-P44/42 MAPK | 4370T | 1:2000 | CST |
| P44/42 MAPK | 4695T | 1:1000 | CST |
| p-P38 MAPK | 4511T | 1:1000 | CST |
| P38 MAPK | 8690T | 1:1000 | CST |
| p-JNK | 4668T | 1:1000 | CST |
| MAPK/JNK | 9252T | 1:1000 | CST |
| α-SMA | MAB1420 | 1:1000 | R&D Systems |
| Fibronectin | ab2413 | 1:1000 | Abcam |
| Tubulin | Ab7251 | 1:2000 | Abcam |
| GAPDH | Ab8245 | 1:1000 | Abcam |
| GPER1 | Ab39742 | 1μg/ml | Abcam |
| F4/80 | Ab111101 | 1:100 | Abcam |
| CD86 | Ab239075 | 1:100 | Abcam |
| CD206 | Ab64693 | 0.1μg/ml | Abcam |
| CD68 | Ab955 | 1:50 | Abcam |

**Supplementary Table 4. Antibodies for flow cytometry analysis**

| **Antibodies** | **Catalog No.** | **Company** |
| --- | --- | --- |
| PE-Cy7 Rat Anti-Mouse CD86 | 560582 | BD Pharmingen |
| Alexa Fluor 647 Rat Anti-Mouse CD206 | 565250 | BD Pharmingen |
| APC-Cy7 Mouse Anti-Mouse CD45 | 560694 | BD Pharmingen |
| FITC Rat Anti-CD11b | 557396 | BD Pharmingen |
| BV421 Rat Anti-Mouse F4/80 | 565411 | BD Pharmingen |

**Supplementary original western blots**

**
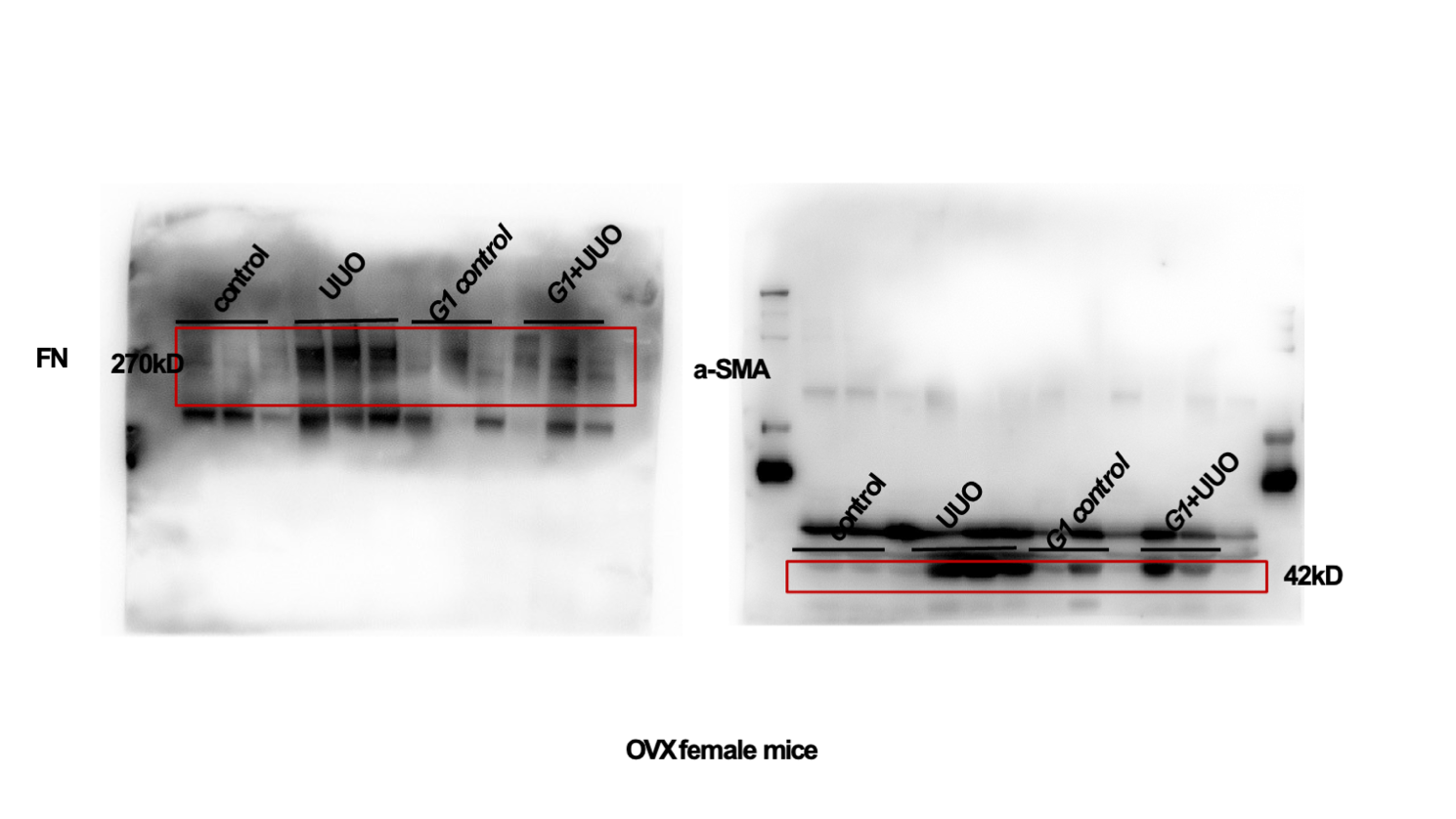
**

**
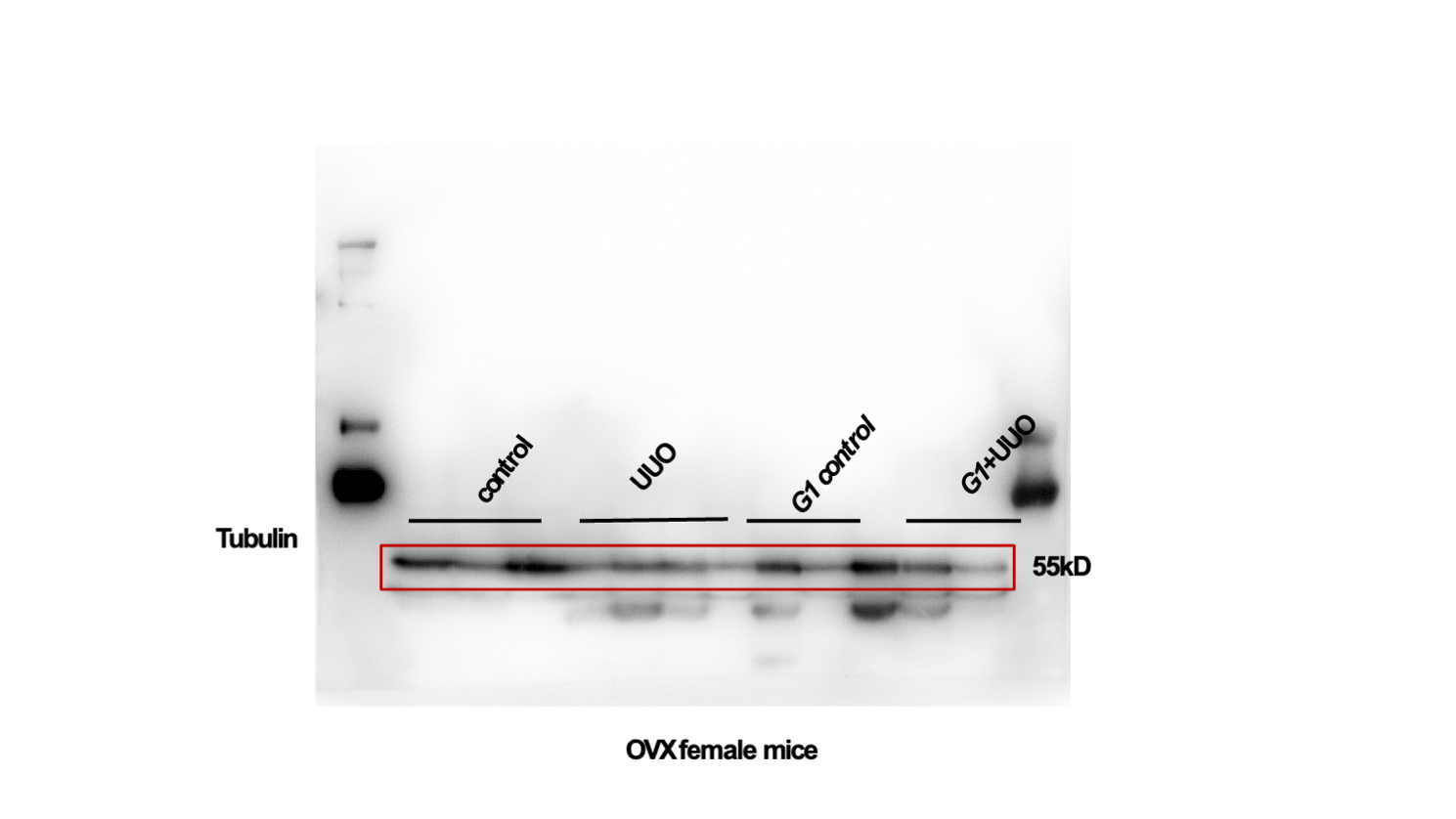
**

**
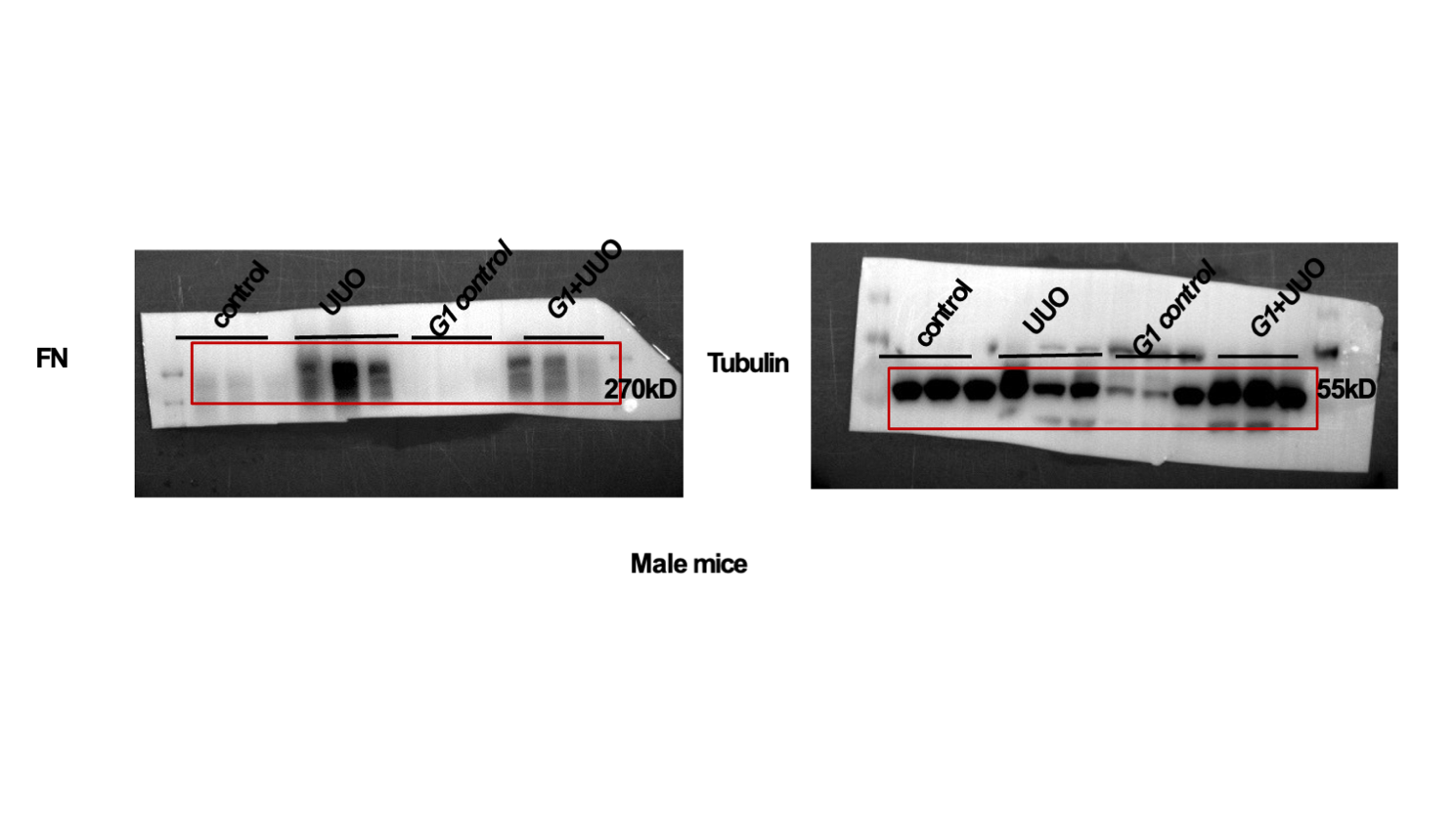
**

**
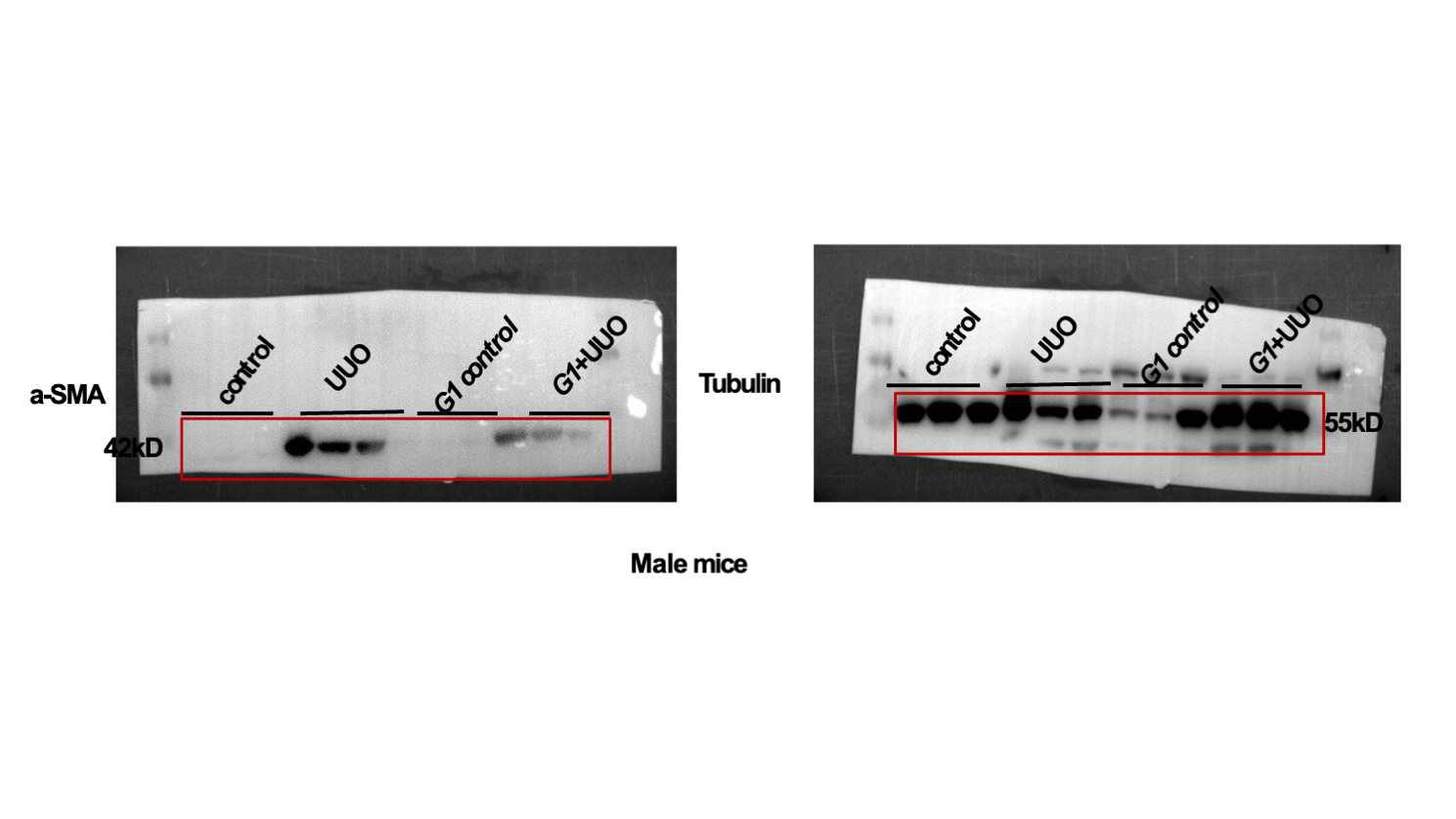
**

**
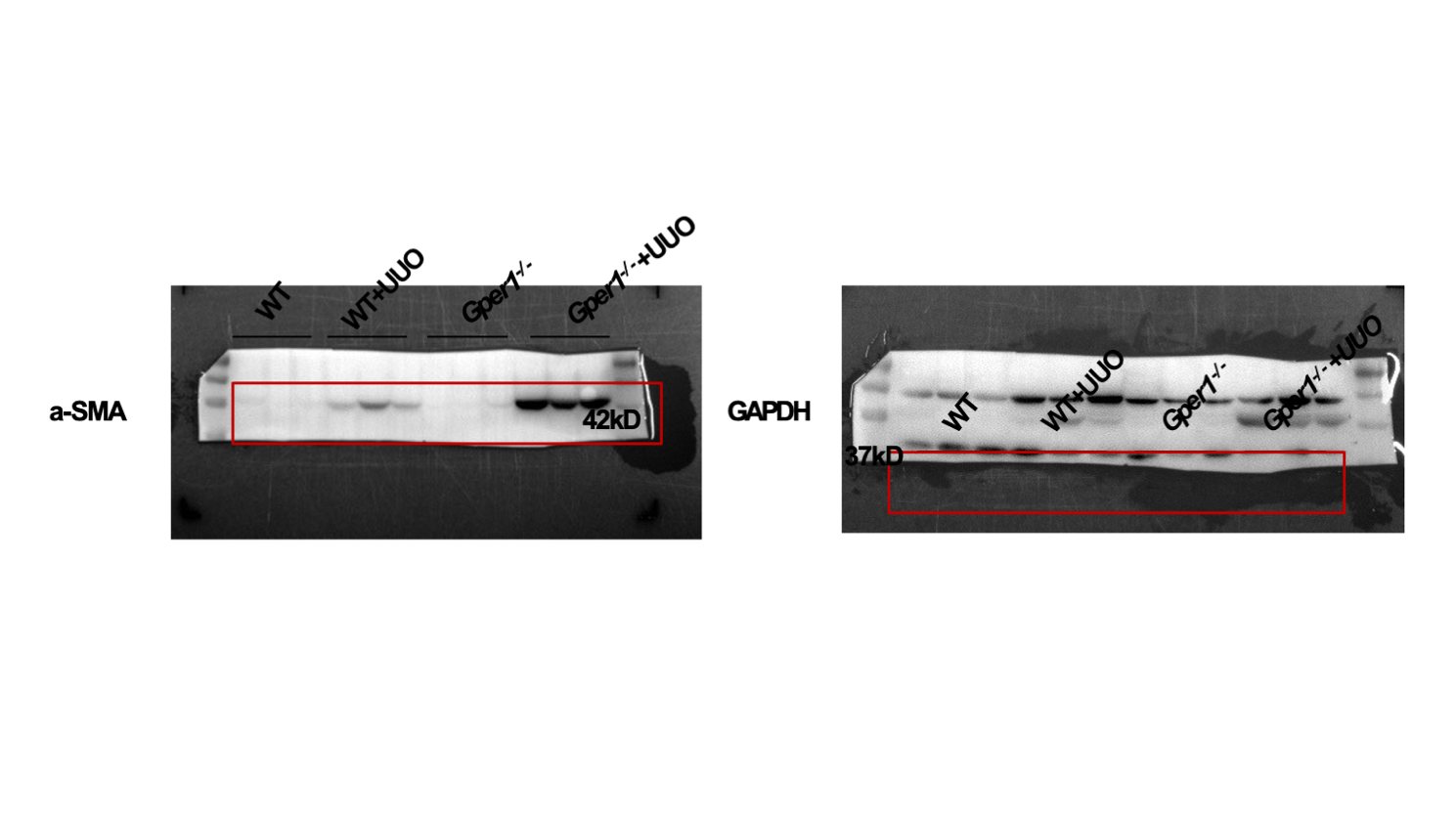
**

**
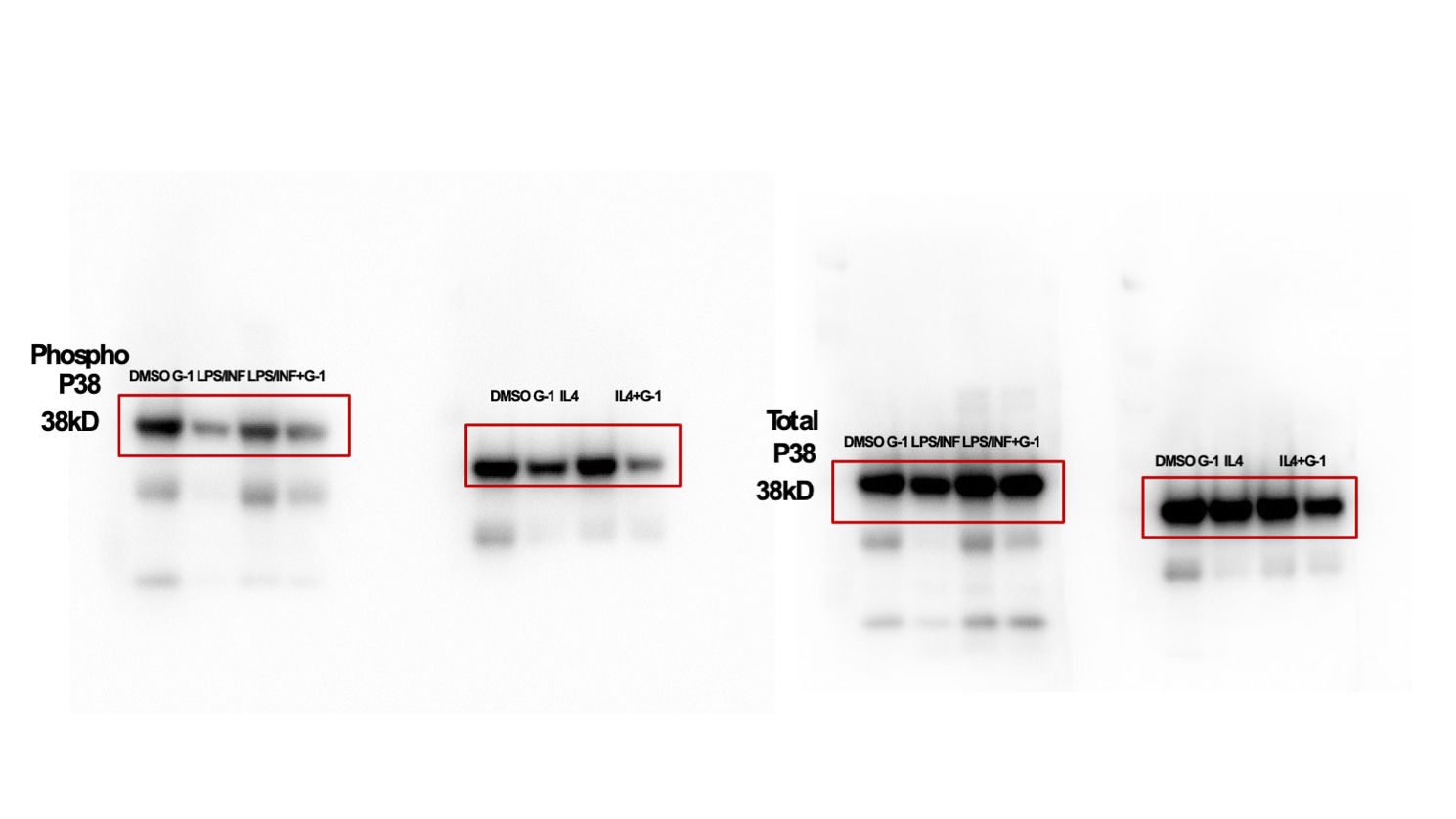
**

**
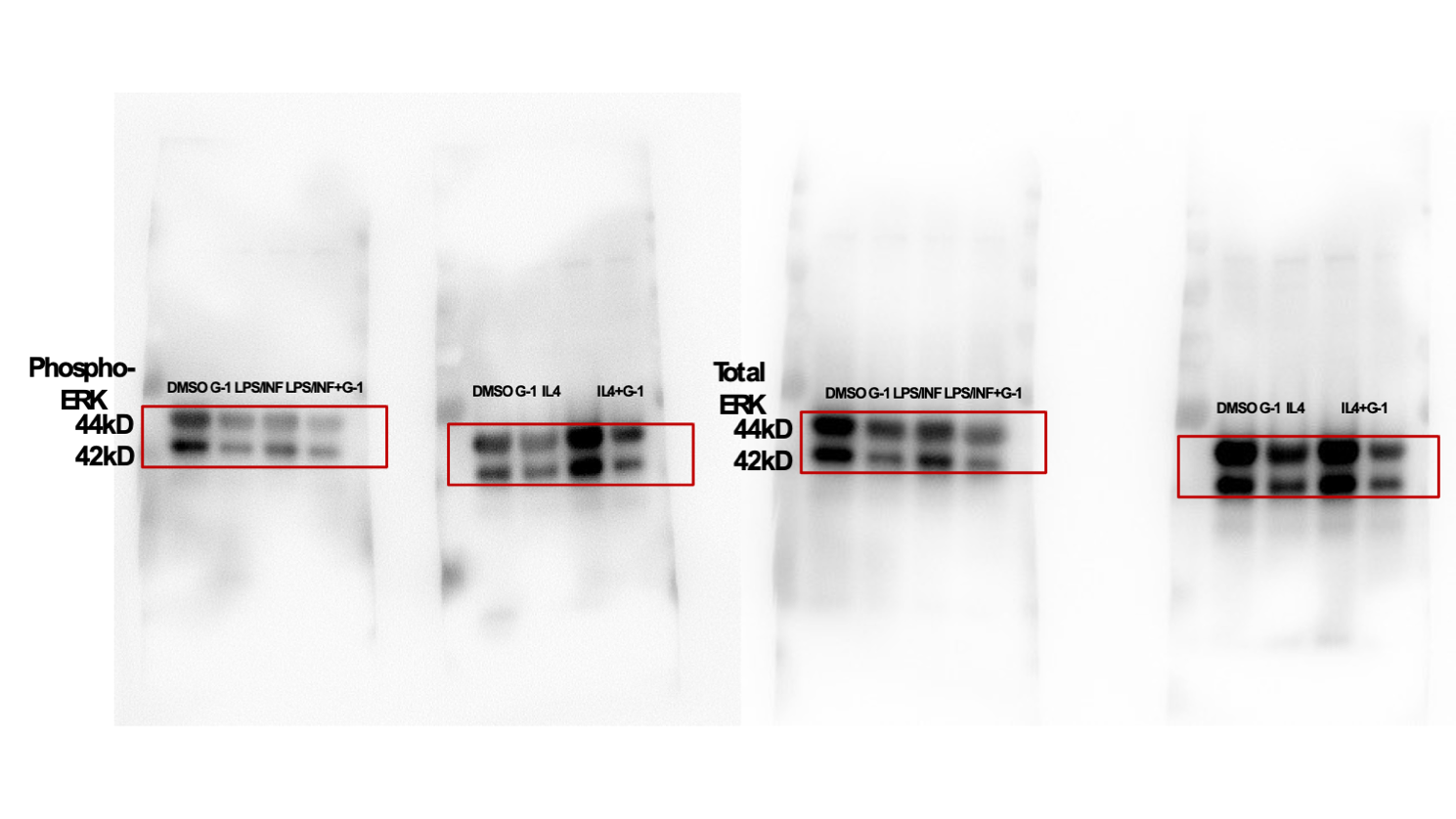
**

**
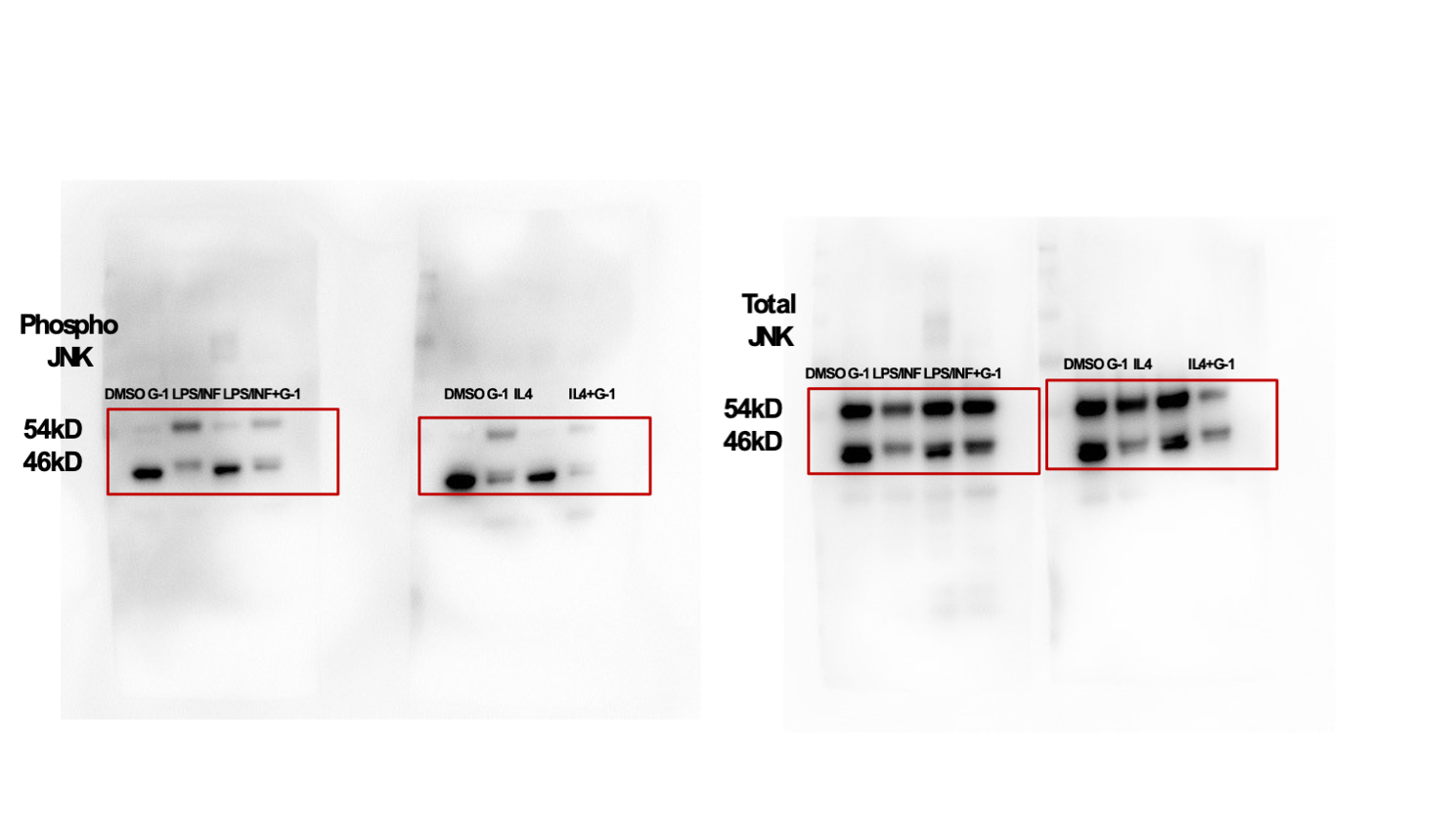
**

**
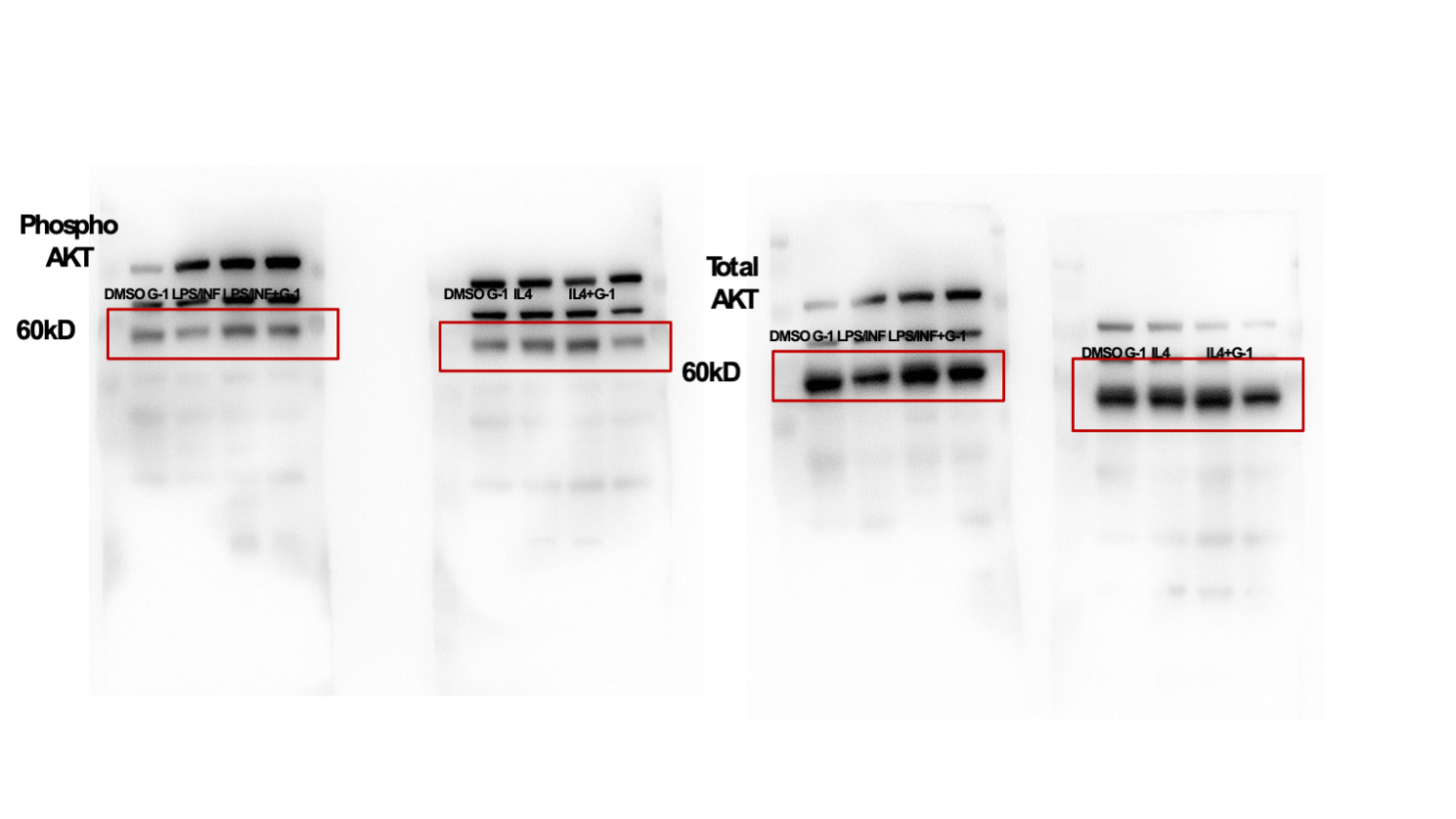
**

**
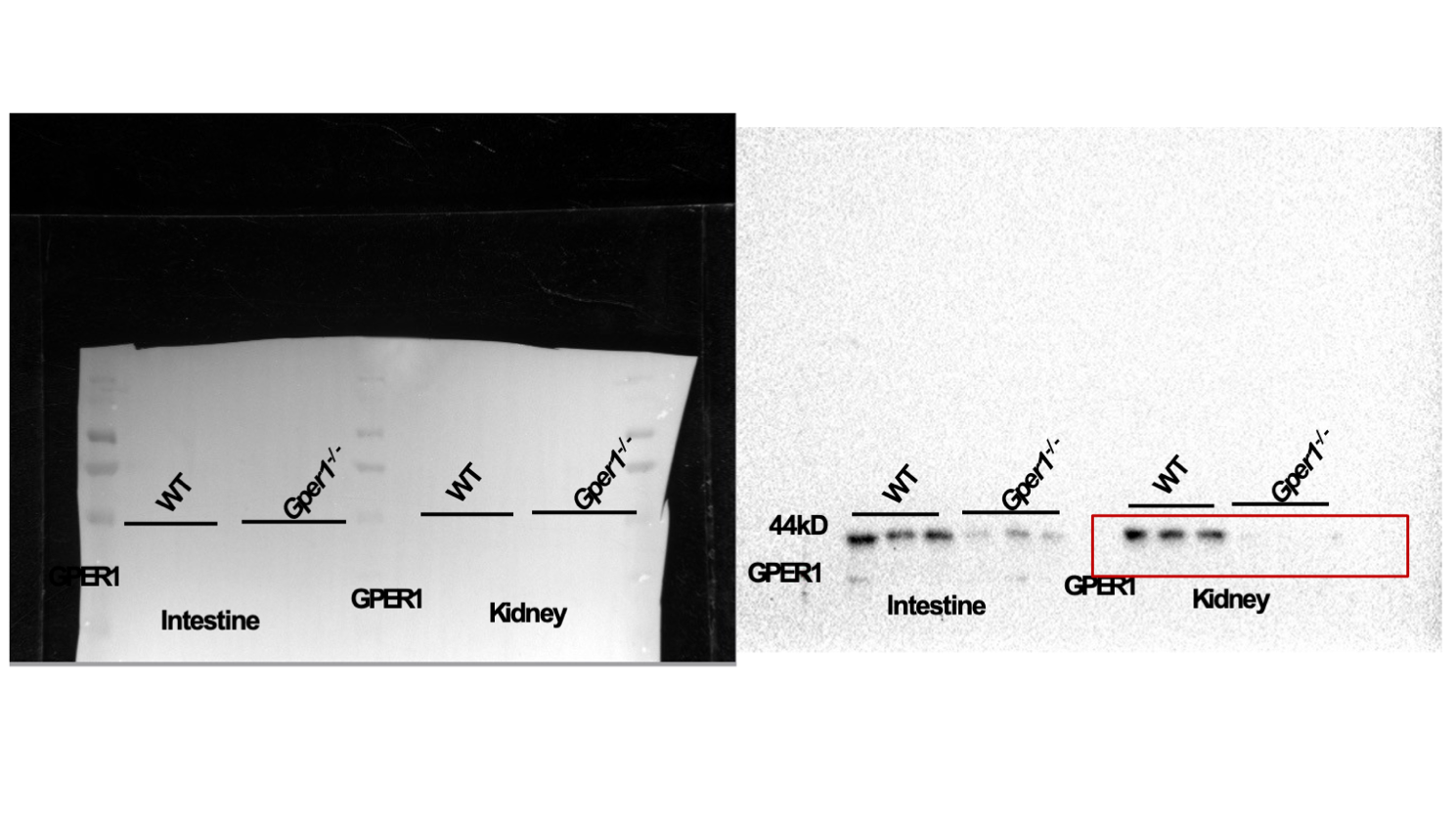
**

**
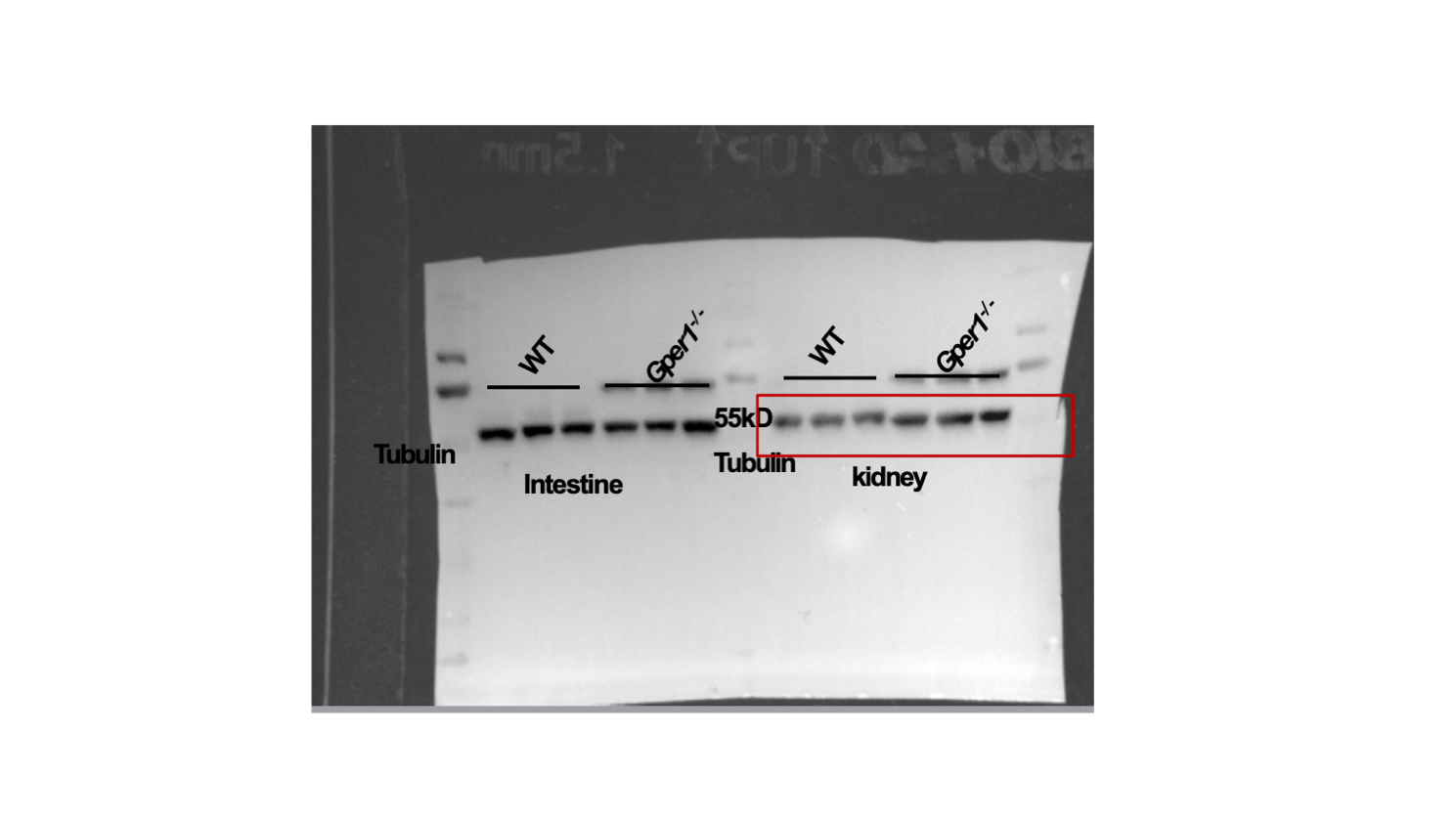
**
