# Supplementary material for: Activation of GPER1 in macrophages ameliorates UUO-induced renal fibrosis
Source: Cell Death Dis. 2023 Dec 12;14(12):818. doi: 10.1038/s41419-023-06338-2 (PMC10716282; doi:10.1038/s41419-023-06338-2)
Supplement: Supplementary file 2 — Original Data File-WB [file 41419_2023_6338_MOESM2_ESM.pdf]

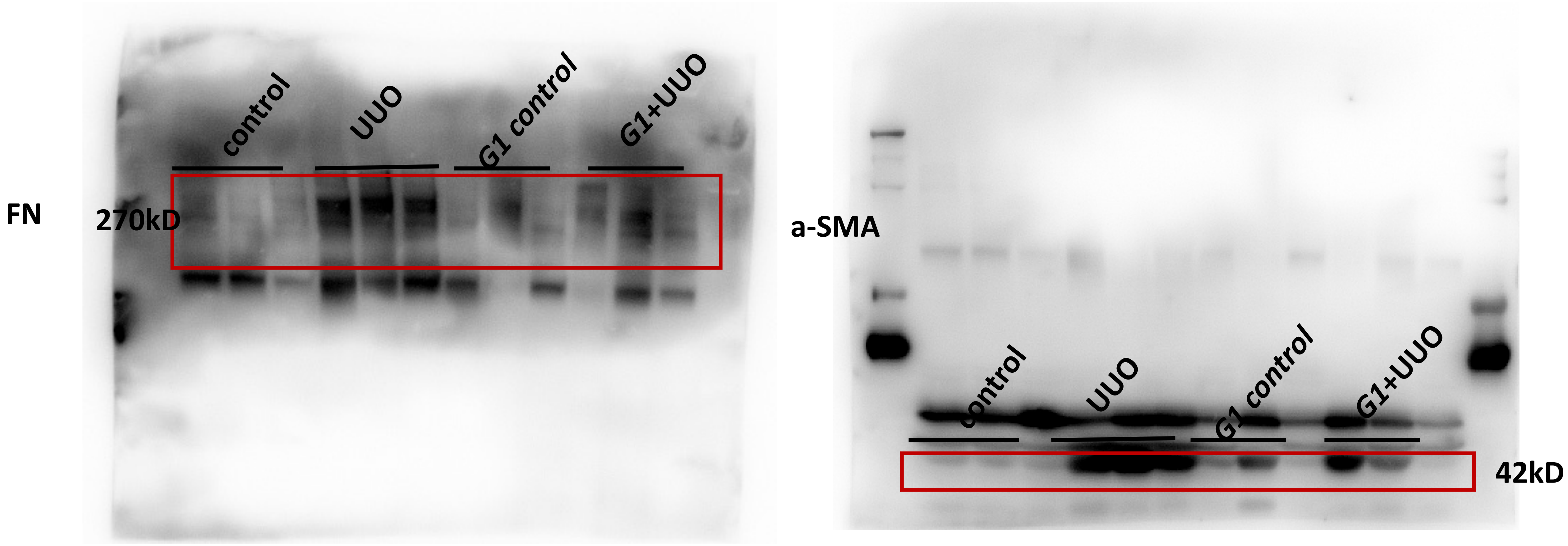

OVX female mice

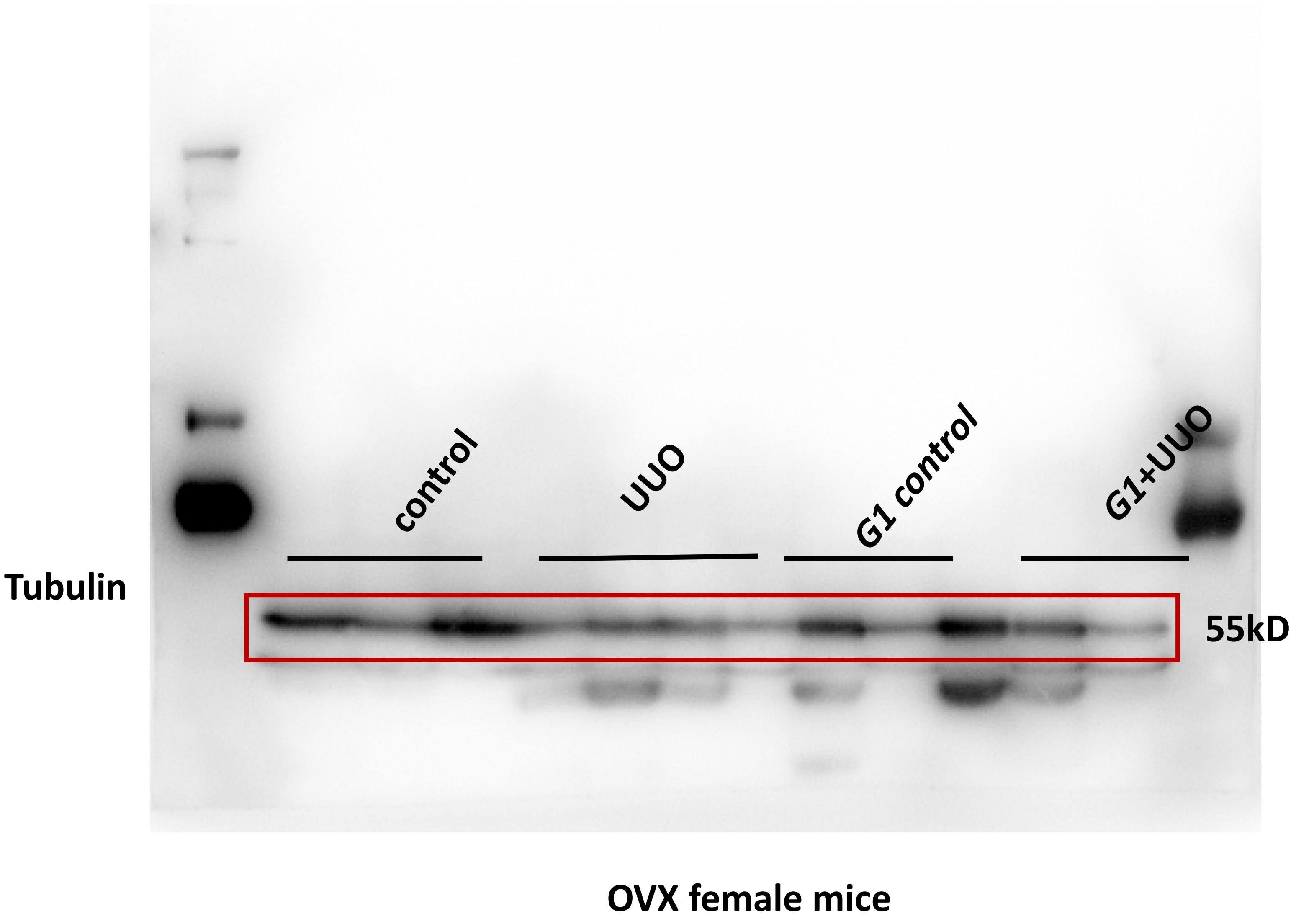
control
UUO
G1 control
G1+UUO
Tubulin
55kD
OVX female mice

FN

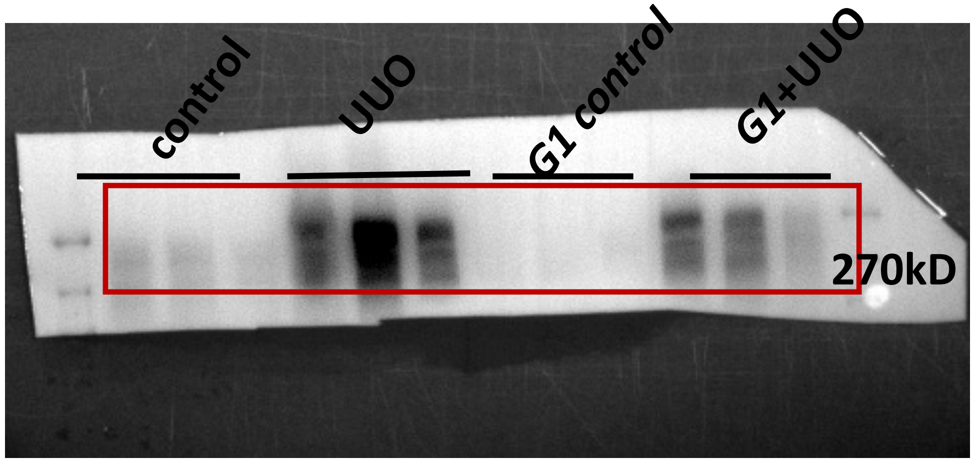

Tubulin

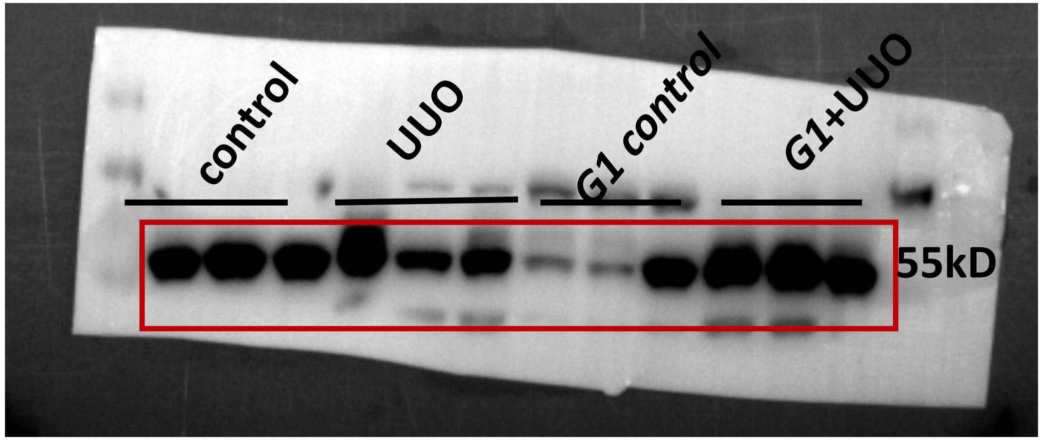

Male mice

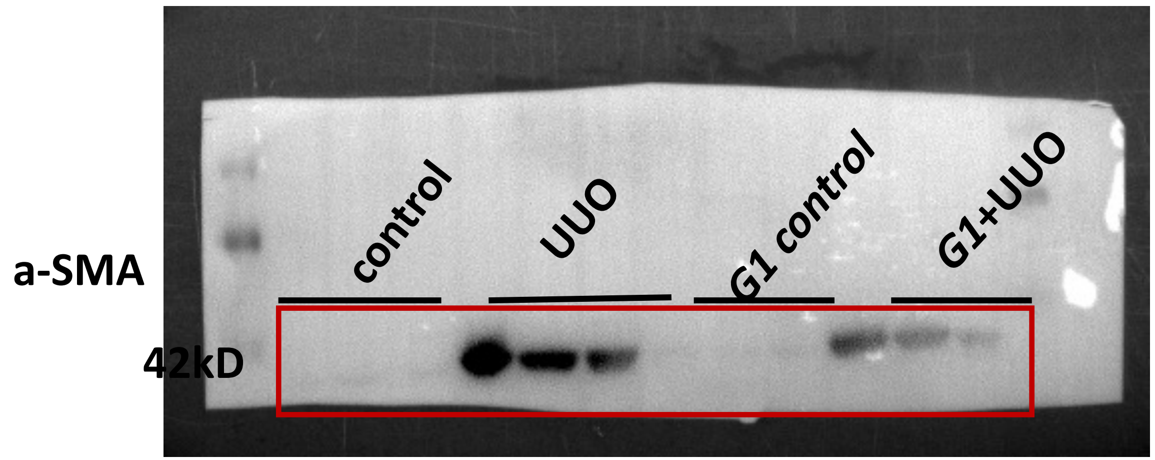
a-SMA
control
UUO
G1 control
G1+UUO
42kD

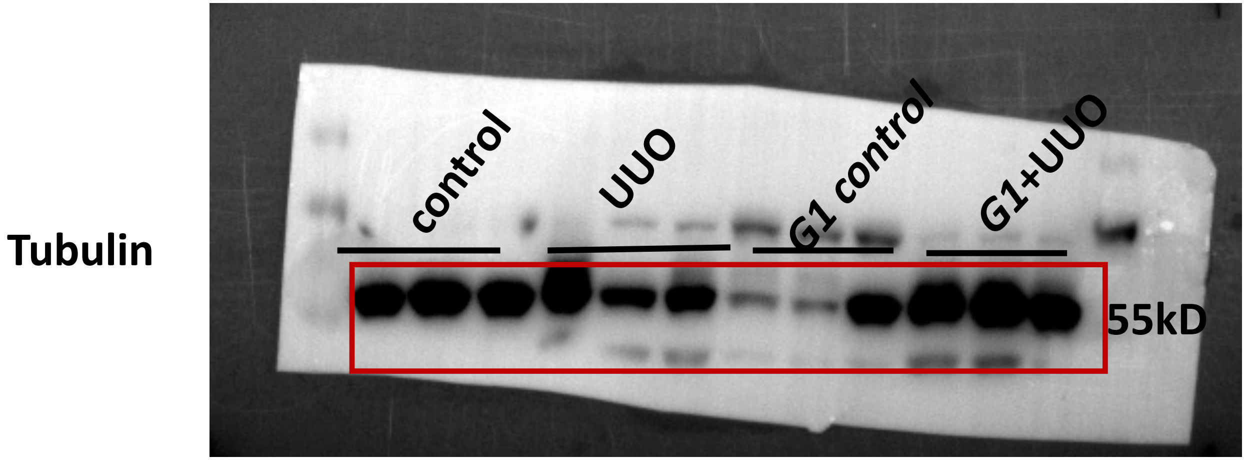
Tubulin
control
UUO
G1 control
G1+UUO
55kD

Male mice

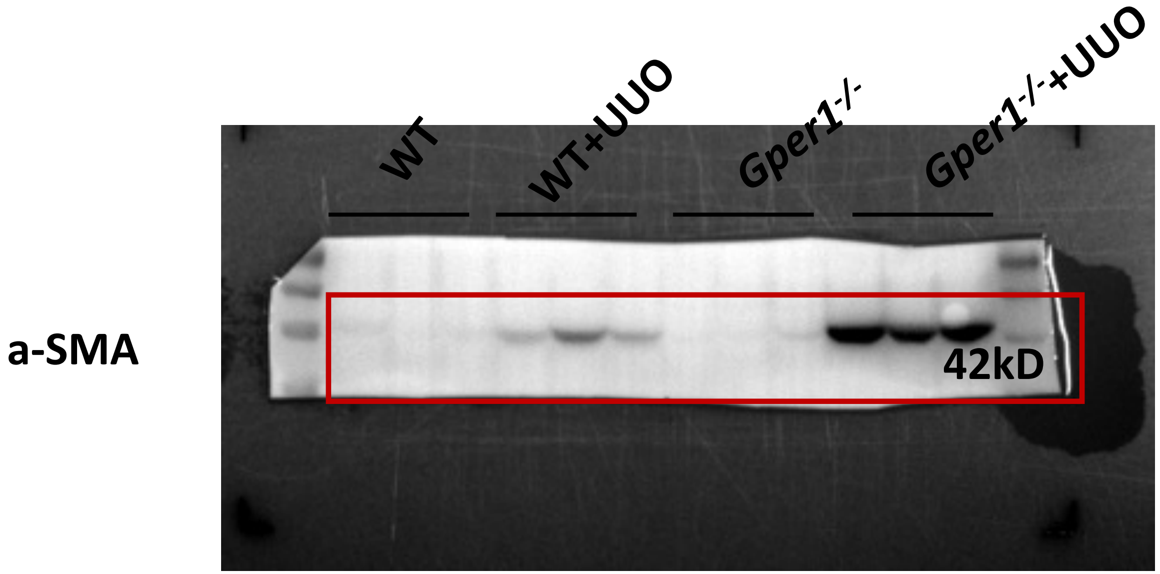
a-SMA
WT
WT+UUO
Gper1-/-
Gper1-/-+UUO
42kD

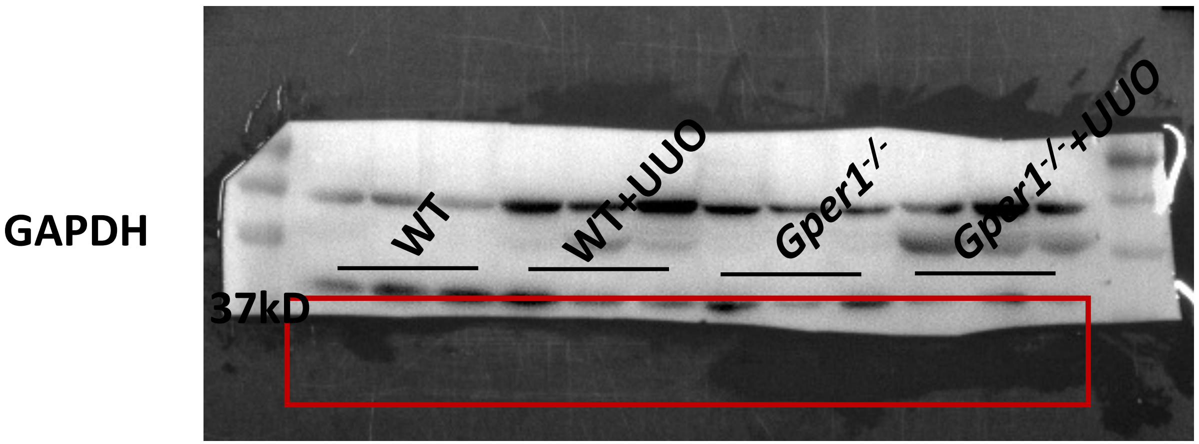
GAPDH
WT
WT+UUO
Gper1-/-
Gper1-/-+UUO
37kD

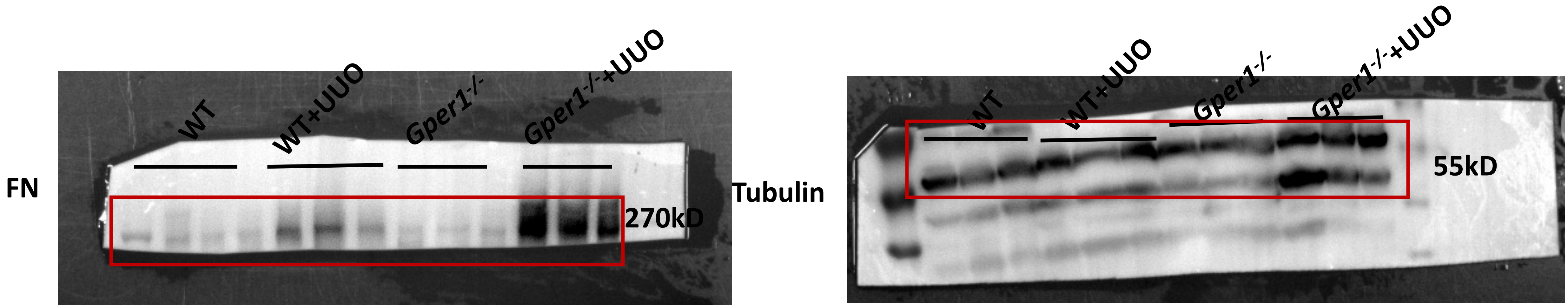
FN
WT
WT+UUO
Gper1-/-
Gper1-/-+UUO
270kD
Tubulin
WT
WT+UUO
Gper1-/-
Gper1-/-+UUO
55kD

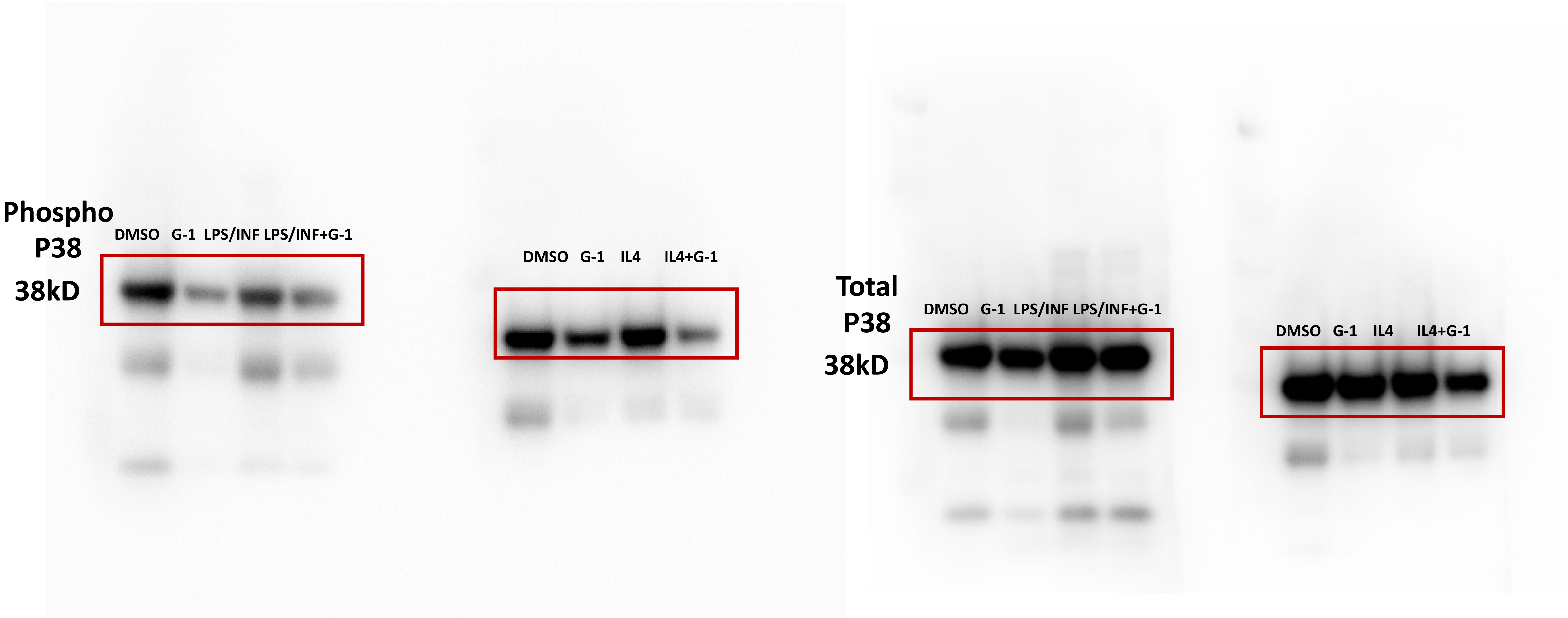

Phospho
P38
38kD
DMSO G-1 LPS/INF LPS/INF+G-1
DMSO G-1 IL4 IL4+G-1
Total
P38
38kD
DMSO G-1 LPS/INF LPS/INF+G-1
DMSO G-1 IL4 IL4+G-1

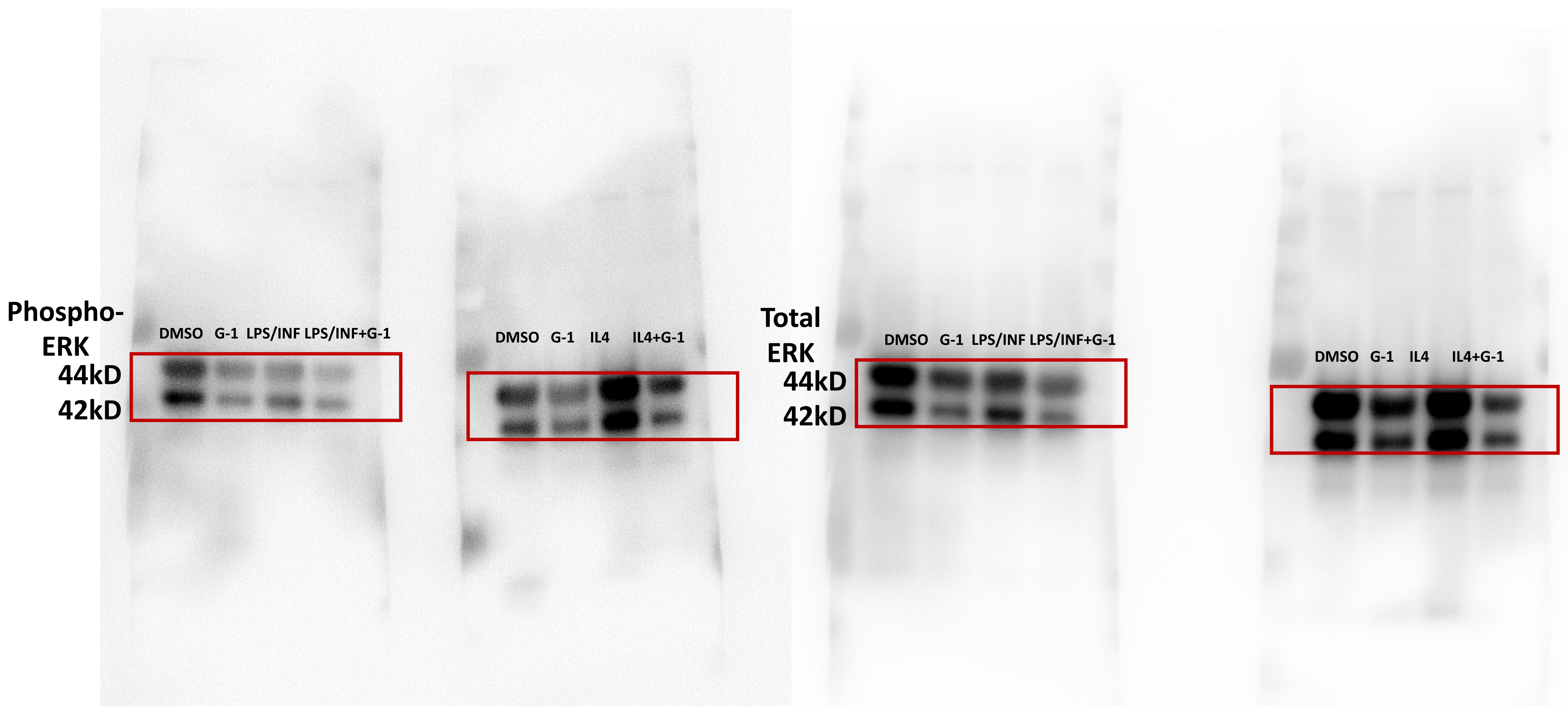

Phospho-ERK
44kD
42kD
DMSO G-1 LPS/INF LPS/INF+G-1
DMSO G-1 IL4 IL4+G-1
Total ERK
44kD
42kD
DMSO G-1 LPS/INF LPS/INF+G-1
DMSO G-1 IL4 IL4+G-1

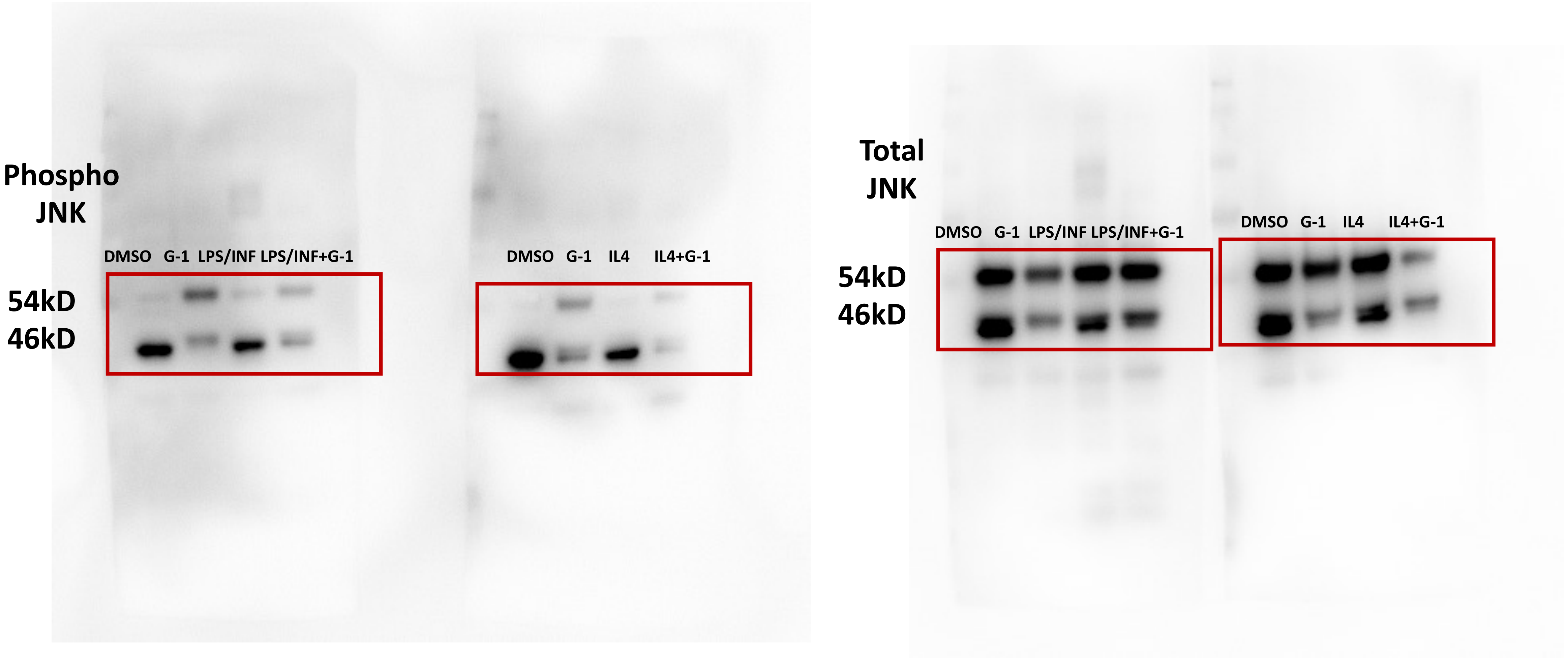
Phospho JNK
DMSO G-1 LPS/INF LPS/INF+G-1
DMSO G-1 IL4 IL4+G-1
54kD
46kD
Total JNK
DMSO G-1 LPS/INF LPS/INF+G-1
DMSO G-1 IL4 IL4+G-1
54kD
46kD

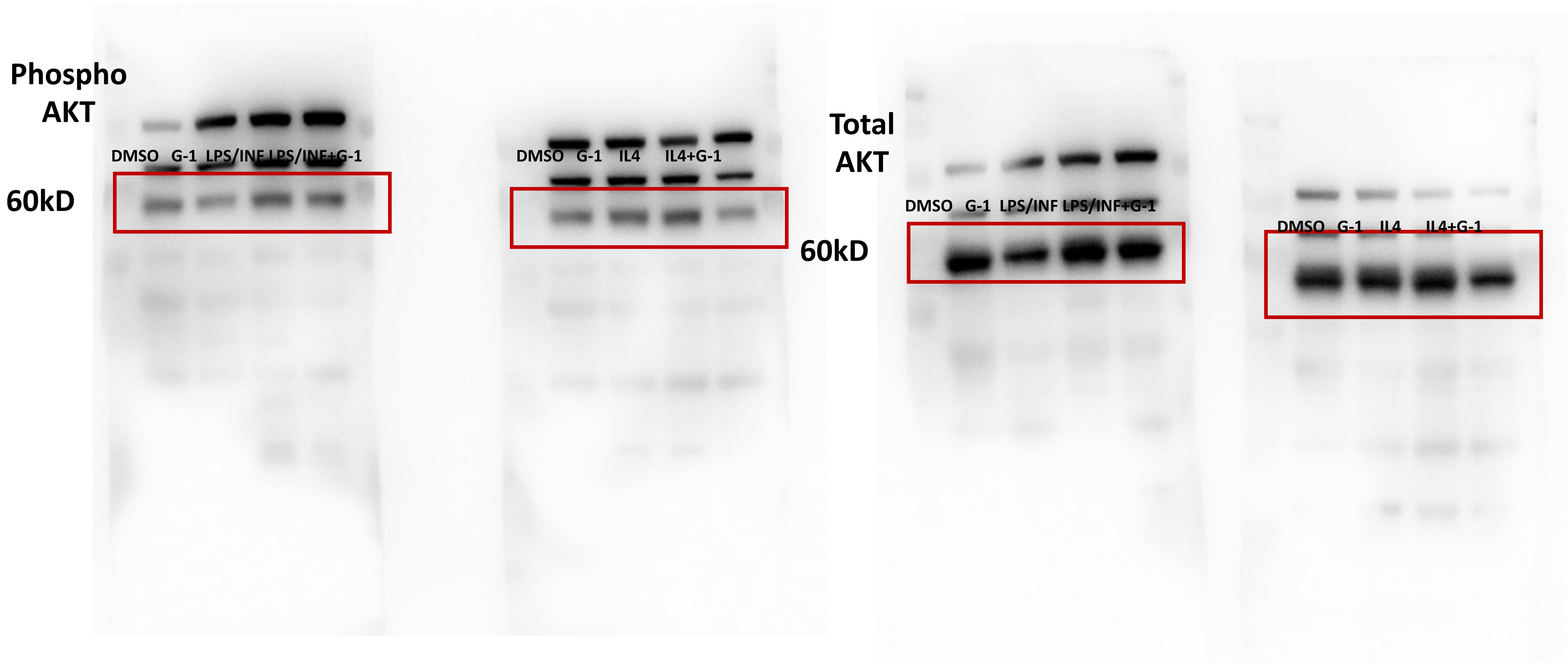
Phospho AKT
DMSO G-1 LPS/INF LPS/INF+G-1
60kD
DMSO G-1 IL4 IL4+G-1
Total AKT
DMSO G-1 LPS/INF LPS/INF+G-1
60kD
DMSO G-1 IL4 IL4+G-1

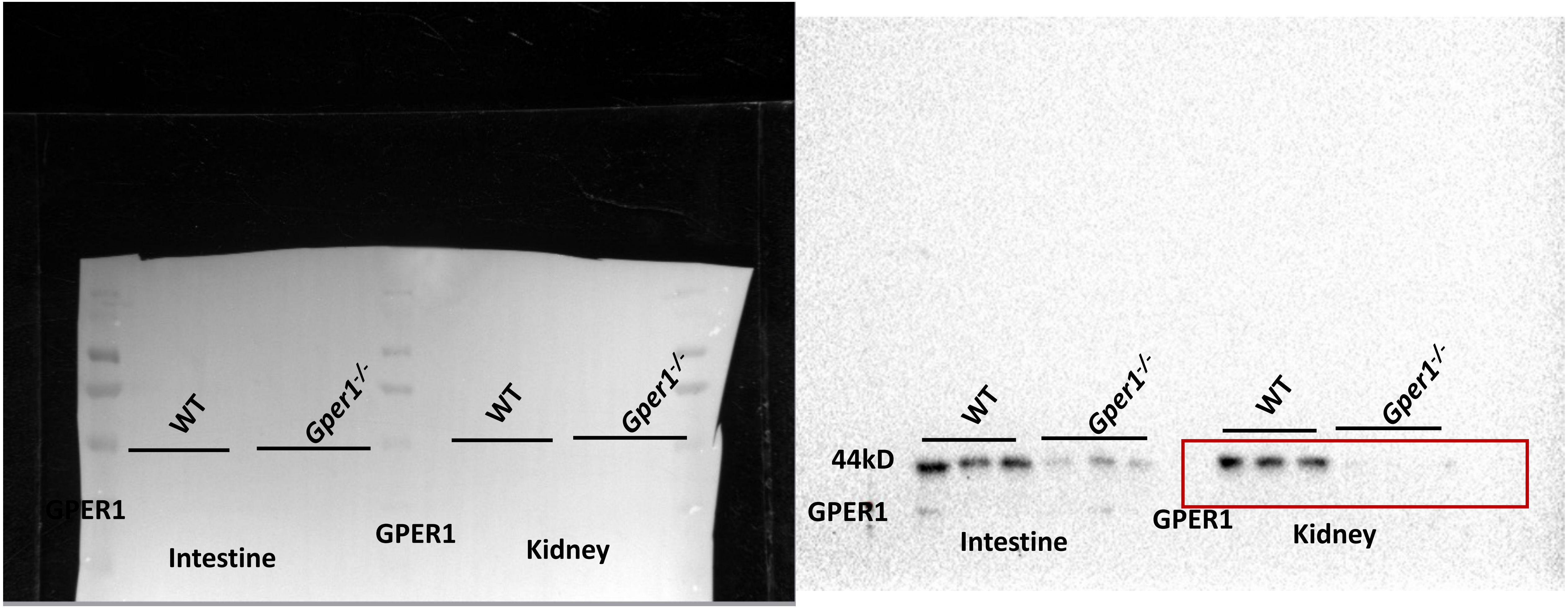
WT
Gper1-/-
WT
Gper1-/-
GPER1
GPER1
Intestine
Kidney
44kD
WT
Gper1-/-
WT
Gper1-/-
GPER1
Intestine
GPER1
Kidney

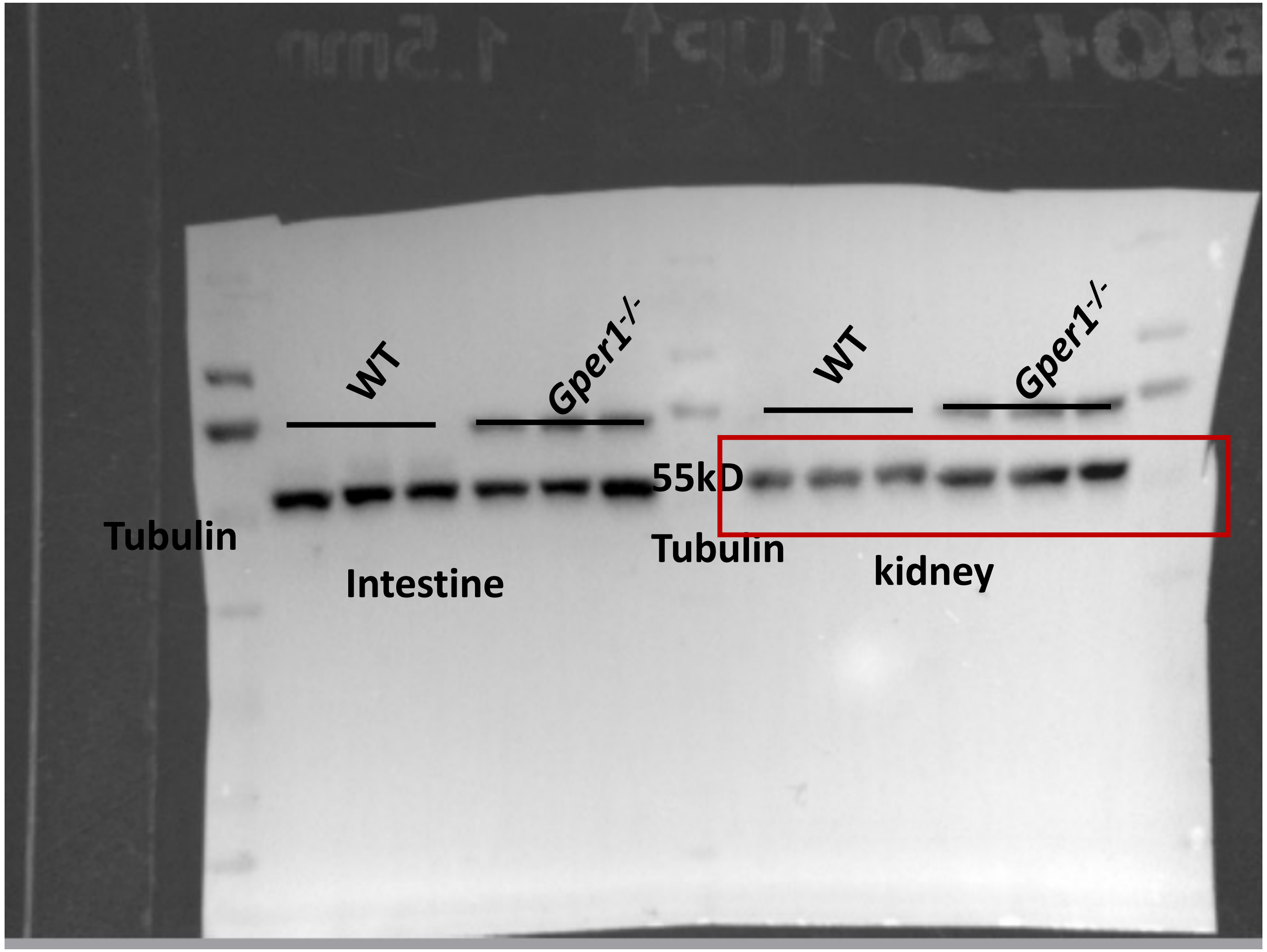
WT
Gper1-/-
Tubulin
Intestine
55kD
Tubulin
WT
Gper1-/-
kidney
